# Supplementary material for: Integrating antigen capturing nanoparticles and type 1 conventional dendritic cell therapy for in situ cancer immunization
Source: Nat Commun. 2025 May 16;16:4578. doi: 10.1038/s41467-025-59840-w (PMC12084569; doi:10.1038/s41467-025-59840-w)
Supplement: Supplementary file 1 — Supplementary Information [file 41467_2025_59840_MOESM1_ESM.pdf]

Supplementary Materials for

Integrating antigen capturing nanoparticles and type 1 conventional dendritic cell therapy for *in situ* cancer immunization

Chih-Jia Chao *et al.*

*Corresponding author. Email: zhaozm@uic.edu

This PDF file includes:

Supplementary Methods
Supplementary Tables
Supplementary Figures S1 to S45

Supplementary Methods

Antibodies and their dilutions used in this work

- CD45-Alexa Fluor 700 (clone QA17A26, BioLegend, catalog #157616), 1:400-1:2000 dilution
- B220-APC (clone RA3-6B2, BioLegend, catalog #103211), 1:300 dilution
- CD11c-APC/Cy7 (clone N418, BioLegend, catalog #117324), 1:50 dilution
- CD11c-PE, (clone N418, BioLegend, catalog #117308), 1:1000 dilution
- CD11c-BV650 (clone N418, BioLegend, catalog #117339), 1:80-1:200 dilution
- CD103-Alexa Fluor 700, (clone 2E7, BioLegend, catalog #121442), 1:200 dilution
- CD103-BV711(clone 2E7, BioLegend, catalog #121435), 1:100 dilution
- CD86-APC, (clone GL-1, BioLegend, catalog #105012), 1:400 dilution
- CD86-BV785 (clone GL-1, BioLegend, catalog #105043), 1:50-1:200 dilution
- MHCII-PB, (clone M5/114.15.2, BioLegend, catalog #107620), 1:1000 dilution
- MHCII-BV605 (clone M5/114.15.2, BioLegend, catalog #107639), 1:50 dilution
- CD80-FITC, (clone 16-10A1, BioLegend, catalog #104706), 1:250 dilution
- CD80-BV605 (clone 16-10A1, BioLegend, catalog #104729), 1:20-1:40 dilution
- CD11b-FITC (clone M1/70, BioLegend, catalog #101205), 1:100-1:1000 dilution
- F4/80-BV510 (clone BM8, BioLegend, catalog #123135), 1:10-1:200 dilution
- CD206-Percp-Cyanine5.5 (clone C068C2, BioLegend, catalog #141715), 1:10-1:200 dilution
- Gr-1-Pacific Blue (clone RB6-8C5, BioLegend, catalog #108430), 1:50 dilution
- CD49b-PE/Dazzle 594 (clone DX5, BioLegend, catalog #108924), 1:100-1:500 dilution
- CD3-PE/Fire 700 (clone 17A2, BioLegend, catalog #100272), 1:50-1:500 dilution
- CD4-Spark Blue 550 (clone GK1.5, BioLegend, catalog #100474), 1:100-1:400 dilution
- CD8a-Spark UV 387 (clone 53-6.7, BioLegend, catalog #100798), 1:100-1:1000 dilution
- IFN-γ-APC/Fire 750 (clone XMG1.2, BioLegend, catalog #505860), 1:100-1:200 dilution
- CD62L-PE/Cyanine5 (clone MEL-14, BioLegend, catalog #104410), 1:50-1:200 dilution
- CD44-BV570 (clone IM7, BioLegend, catalog #103037), 1:40 dilution
- CD25-PE/Cyanine7 (clone PC61, BioLegend, catalog #102016), 1:20-1:40 dilution
- FoxP3-Alexa Fluor 647 (clone MF-14, BioLegend, catalog #126408), 1:100-1:200 dilution
- PD-1-BV421 (clone 29F.1A12, BioLegend, catalog #135221), 1:100-1:200 dilution
- Adpgk tetramer-PE (Sequence: ASMTNMELM, Tetramer Core of the National Institutes of Health), 1:100 dilution
- OVA tetramer-PE (Sequence: SIINFEKL, Tetramer Core of the National Institutes of Health), 1:100 dilution
- TRP-2 tetramer-PE (Sequence: SVYDFFVWL, Tetramer Core of the National Institutes of Health), 1:100 dilution
- Rpl18 tetramer-BV421 (Sequence: KILTFDRL, Tetramer Core of the National Institutes of Health), 1:100 dilution
- Granzyme B-PE/Cy7 (clone QA16A02, BioLegend, catalog # 372213) 1:40 dilution
- Lag3-PE/Dazzle594 (clone C9B7W, BioLegend, catalog #125223) 1:80 dilution
- Tim3-PE/Fire640 (clone RMT3-23, BioLegend, catalog # 119749) 1:40-1:80 dilution
- PD1-PE/Fire810 (clone 29F.1A12, BioLegend, catalog #135253) 1:160 dilution
- Perforin-APC/Fire750 (clone S16009A, BioLegend, catalog #154317) 1:40-1:80 dilution
- Ki67-BV711 (clone 11F6, BioLegend, catalog #151227) 1:300 dilution
- Clec9A-PE (clone 7H11, BioLegend, catalog #143504), 1:150 dilution
- H-2Kb-SIINFEKL-PE (clone 25-D1.16, BioLegend, catalog #141603), 1:160 dilution
- Zombie NIR (BioLegend, catalog #423106), 1:1000 dilution
- Zombie UV (BioLegend, catalog #423107), 1:1000 dilution

Supplementary Tables

Supplementary Table 1. Encapsulation efficiency and loading efficiency of PIC in nanoparticles.

	**NP^{PEG}**	**NP^{Neg}**	**AC-NP**
Encapsulation efficiency (%)	38.0 ± 8.9	35.5 ± 7.2	85.3 ± 1.9
Loading efficiency (%)	5.7 ± 1.3	5.3 ± 1.1	12.8 ± 2.9

Supplementary Figures

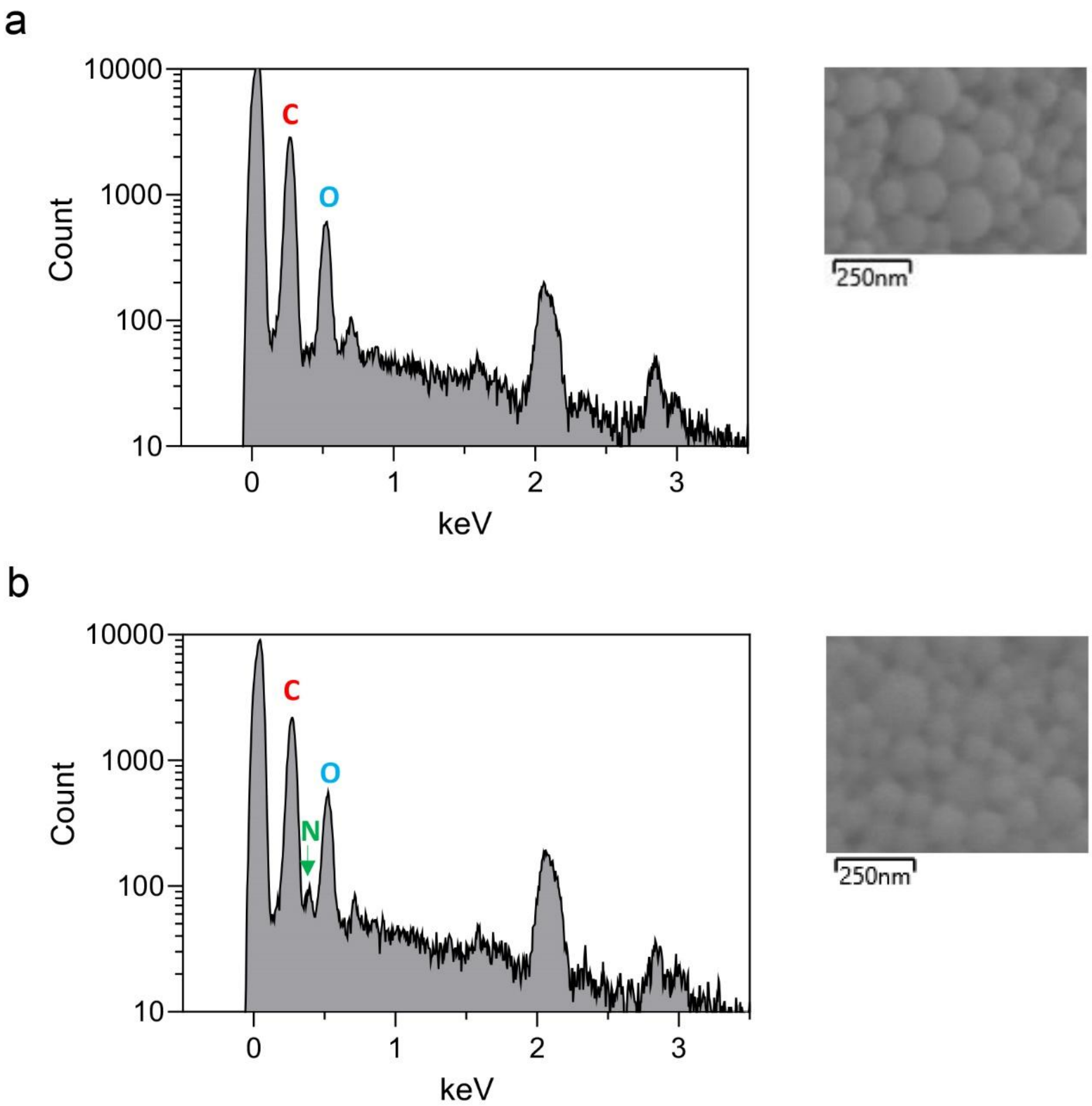

Supplementary Figure 1. EDS profiles of NPs. SEM images and corresponding element spectrum profiles of (**a**) NP^{Neg} (composed of only PLGA) and (**b**) AC-NP (composed of PLGA and PEI) were shown.

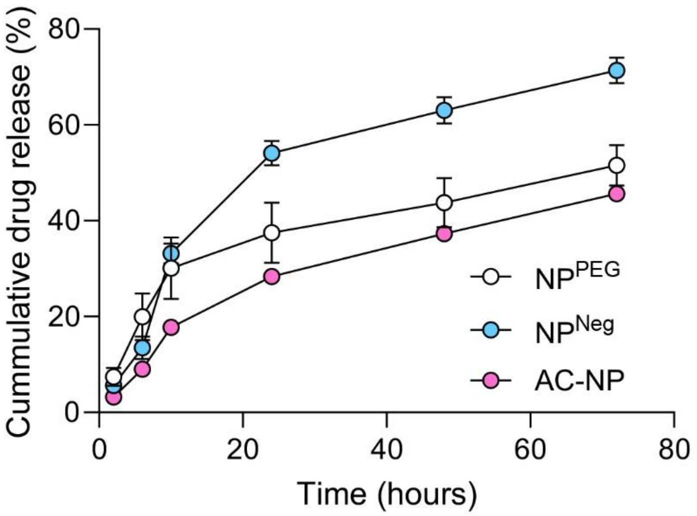

Supplementary Figure 2. Profiles of PIC release from nanoparticles. Release study was conducted in PBS (10 mM, pH 7.4) at 37 ℃. n=3 independent samples for each nanoparticle type. Data are presented as mean values ± SEM. Source data are provided as a Source Data file.

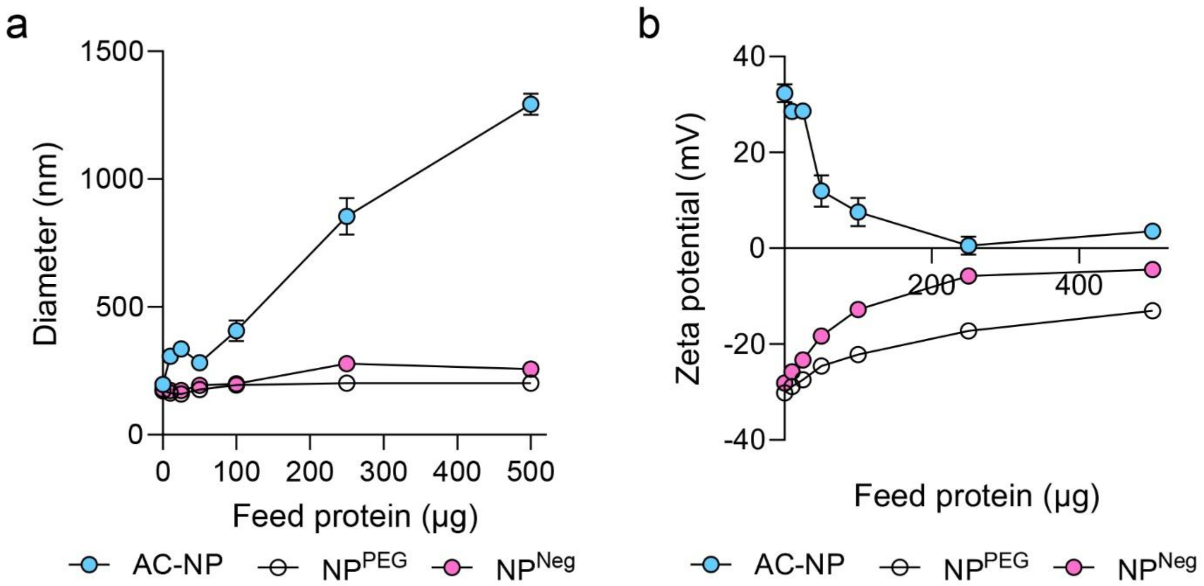

Supplementary Figure 3. Change of size and surface charge of AC-NPs after tumor lysate protein binding. **a,** Change of particle size after incubation with various concentrations of M38 tumor lysate. **b,** Change of zeta potential after incubation with tumor lysate. For **a-b**, n=3 independent samples. For **a-b**, data are presented as mean values ± SEM. Source data are provided as a Source Data file.

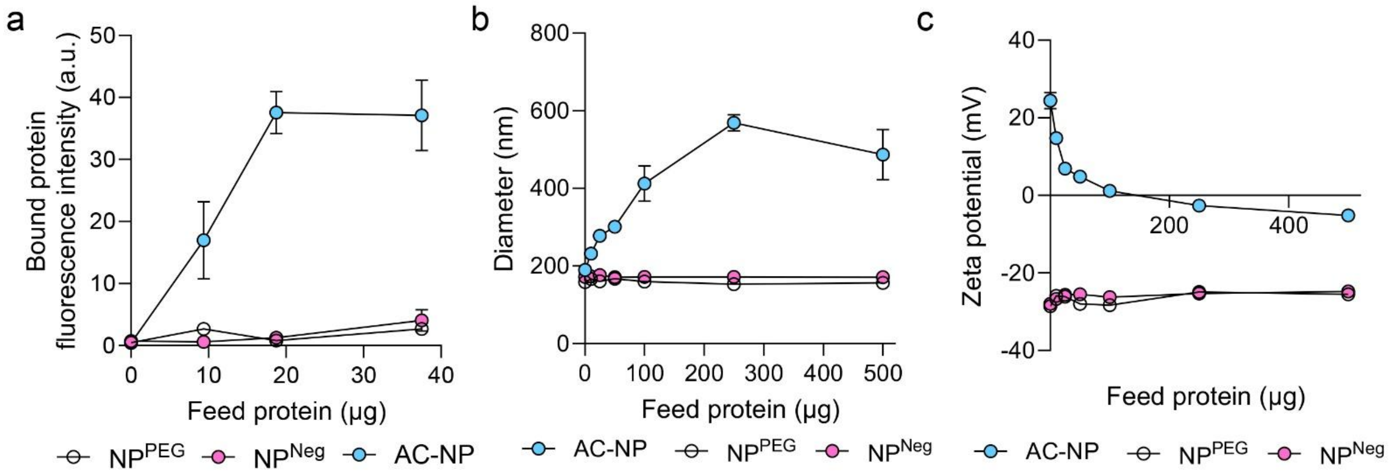

Supplementary Figure 4. Capability of AC-NPs to capture ovalbumin (OVA) and their associated change of particle size and surface charge. a, Relative amount of Alexa Fluor 647-OVA captured by different nanoparticles. **b,** Change of particle size after incubation with various concentrations of OVA. **c,** Change of zeta potential after incubation with OVA. For **a-c**, n=3 independent samples. For **a-c**, data are presented as mean values ± SEM. Source data are provided as a Source Data file.

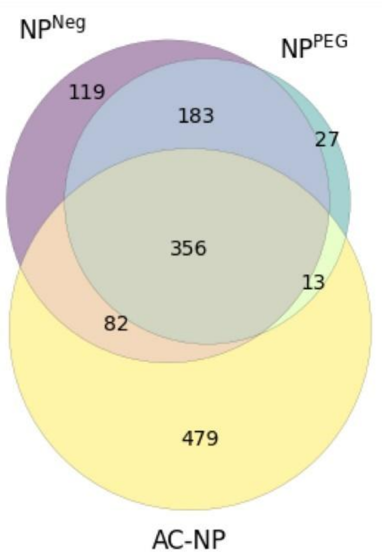

Supplementary Figure 5. Comparison of proteins captured by AC-NPs and control NPs upon incubation with MC38 tumor lysate.

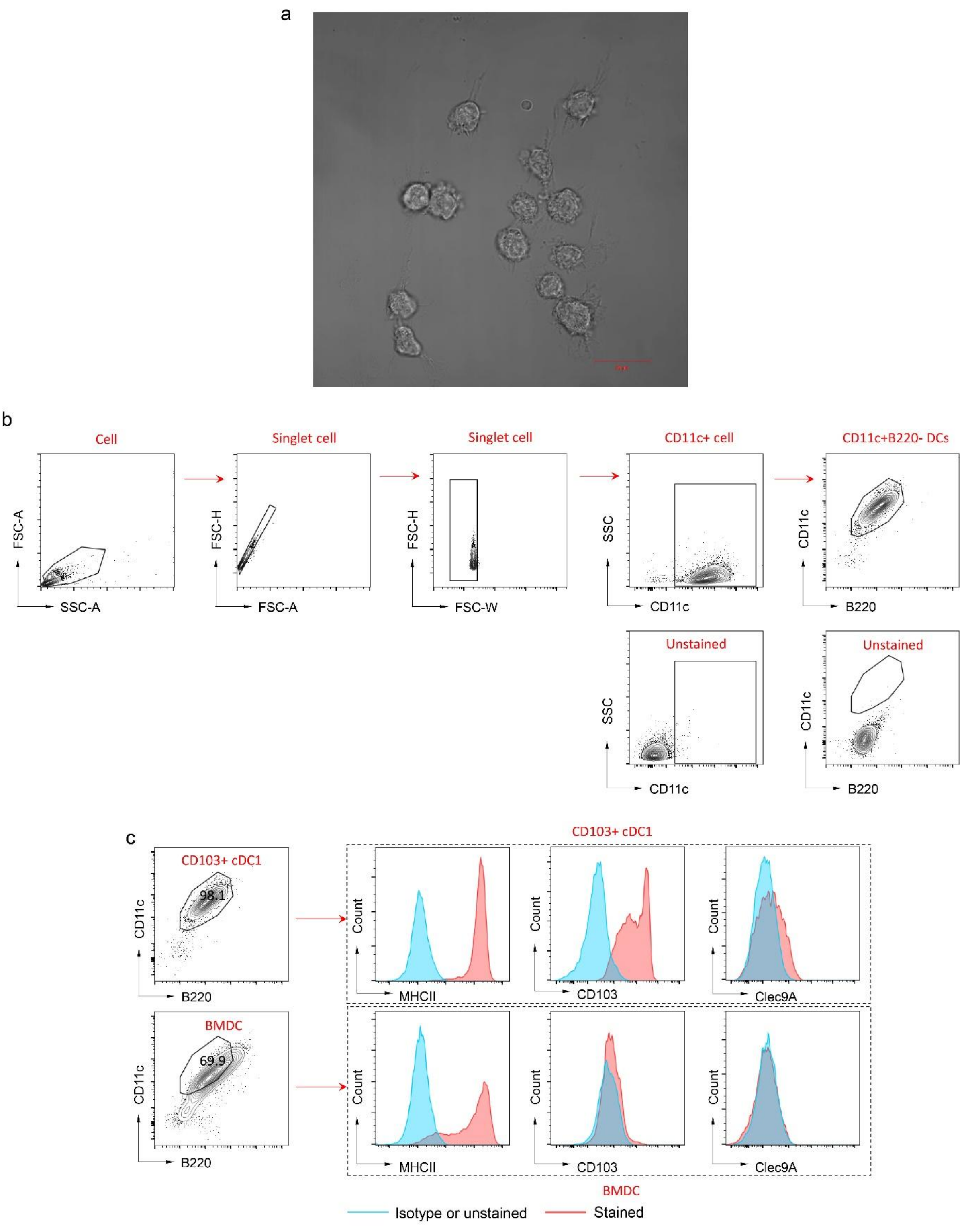

Supplementary Figure 6. Characterization of CD103+ cDC1s. a, Microscopic image of the cultured CD103+ cDC1s. Scale bar: 20 μm. **b**, Representative flow cytometry gating strategy to identify CD11c+B220- DCs. **c**, Representative flow cytometry plots showing that cDC1 characteristic markers including CD103 and Clec9A are overexpressed on the cultured CD103+ cDC1s but not on bone marrow derived DCs (BMDCs).

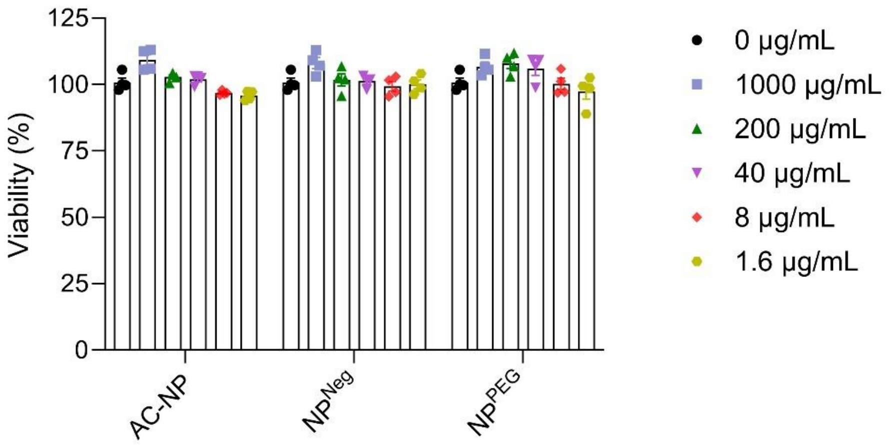

Supplementary Figure 7. Impact of different nanoparticles on the viability of CD103+ cDC1s. CD103+ cDC1s were treated with various concentrations of nanoparticles for 24 hours, and their viability after treatment was measured by a Cell Counting Kit 8 assay. n=3 biologically independent samples. Data are presented as mean values ± SEM. Source data are provided as a Source Data file.

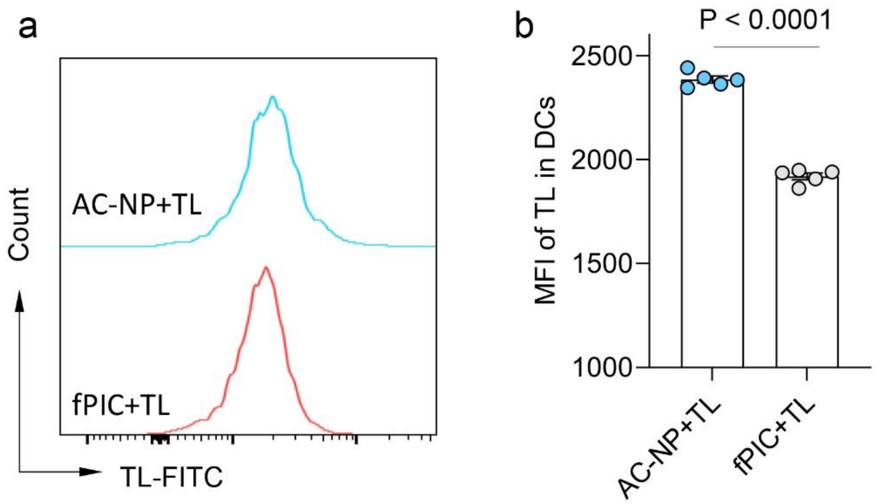

Supplementary Figure 8. AC-NPs enhanced the uptake of tumor lysate into CD103+ cDC1s as compared to cDC1s treated with free PIC. a, Representative flow cytometry plots showing the uptake of FITC-labeled tumor lysate into CD103+ cDC1s. **b**, Relative amount of tumor lysate in CD103+ cDC1s treated by AC-NP or free PIC. For **b**, n=3 biologically independent samples. Data are presented as mean values ± SEM. Statistical analysis was performed using two-tail student's t test. Source data are provided as a Source Data file.

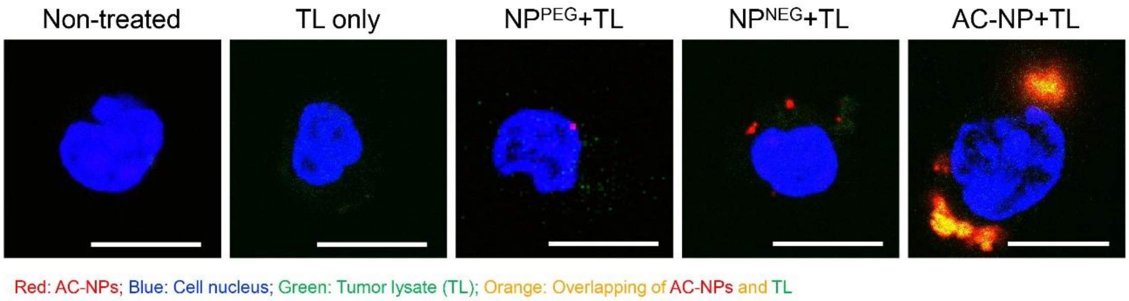

Supplementary Figure 9. Representative confocal microscopic images of cDC1 incubated with MC38 tumor lysates and different nanoparticles for 4 hours. Tumor lysate was labeled by FITC. Nanoparticles were labeled by DiD. Representative of three independent experiments with similar results. Scale bar: 10 μm.

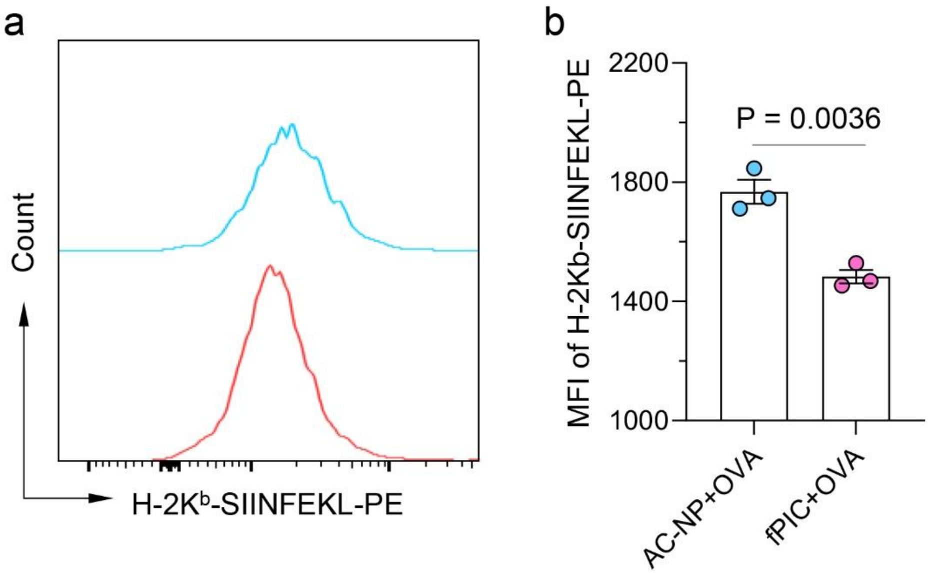

Supplementary Figure 10. AC-NPs enhanced the presentation of OVA peptide SIINFEKL on CD103+ cDC1s as compared to cDC1s treated with free PIC. a, Representative flow cytometry plots showing the expression of SIINFEKL on CD103+ cDC1s. Blue: AC-NP+OVA; Red: free PIC+OVA. **b**, Mean fluorescence intensity of H-2Kb-SIINFEKL-PE on CD103+ cDC1s treated with AC-NP+OVA or free PIC+OVA. For **b**, n=3 biologically independent samples. Data are presented as mean values ± SEM. Statistical analysis was performed using two-tail student's t test. Source data are provided as a Source Data file.

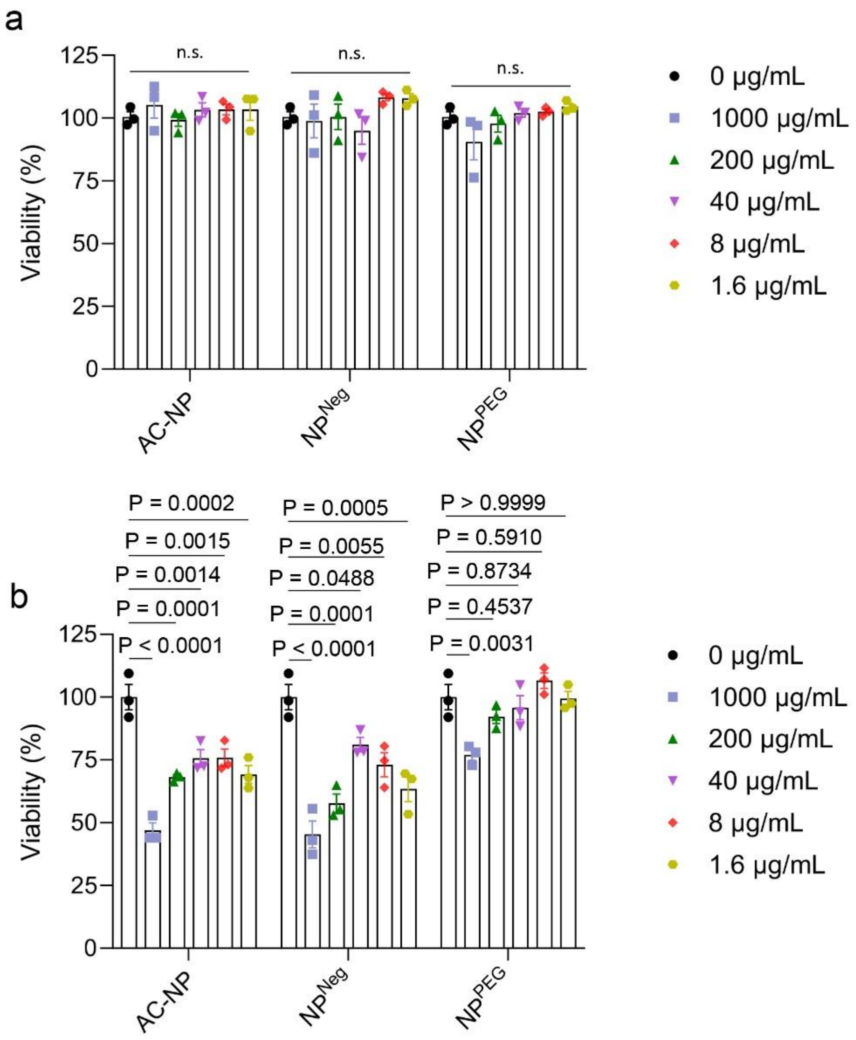

Supplementary Figure 11. Impact of different nanoparticles on the viability of MC38 tumor cells. a, Viability of normal MC38 cells. n.s. indicates no significant difference between the 0 µg/mL group and any other concentration groups (two-way ANOVA followed by Dunnett test). **b**, Viability of MC38 cells pretreated with 1 µg/mL doxorubicin for 12 hours. For **a-b**, n=3 biologically independent samples. For **a-b**, data are presented as mean values ± SEM. Statistical analysis in (**a**, **b**) was performed using one-way ANOVA followed by Dunnett test. n.s.: not significantly different. Normal or doxorubicin-pretreated MC38 tumor cells were co-incubated with various concentrations of nanoparticles for 24 hours, and their viability after treatment was measured by a Cell Counting Kit 8 assay. Source data are provided as a Source Data file.

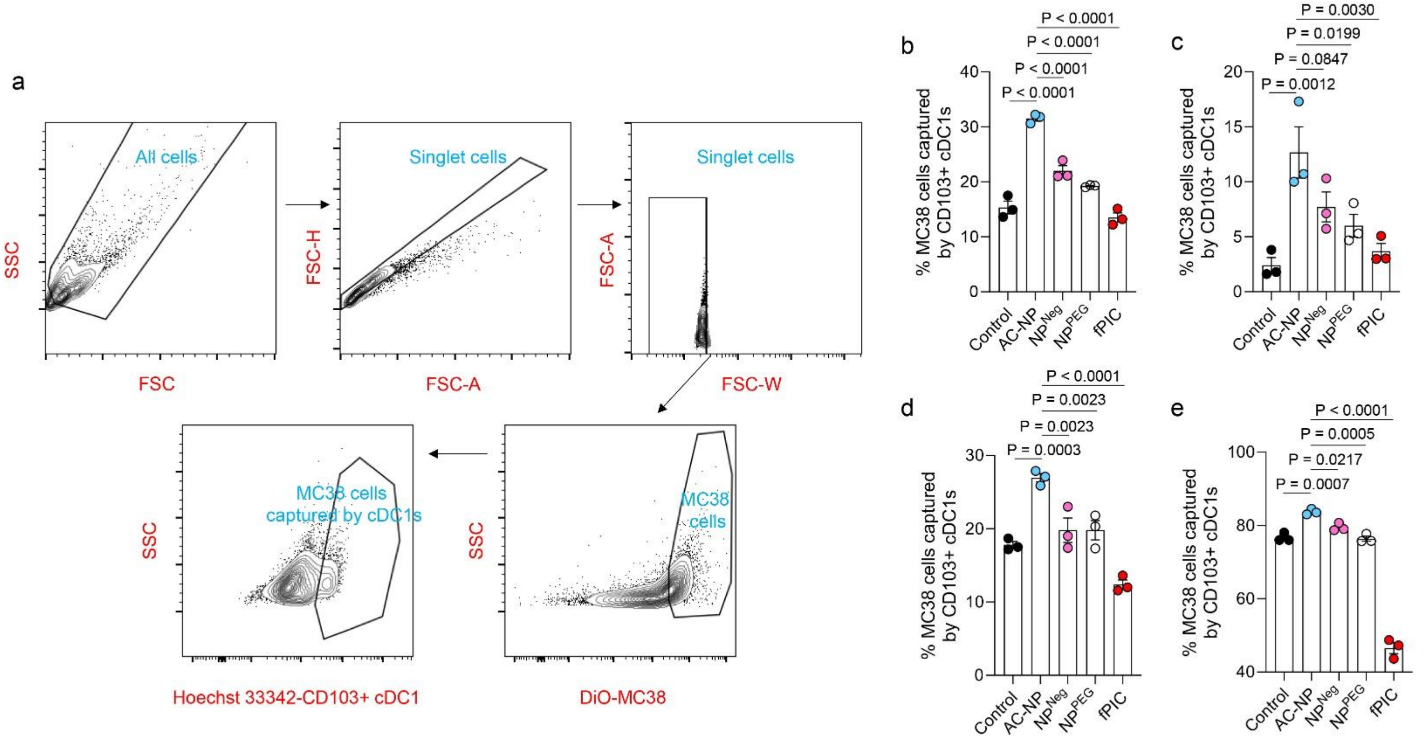

Supplementary Figure 12. AC-NPs enhanced the capture and internalization of MC38 tumor cells into CD103+ cDC1s. a, Representative flow cytometry gating strategy to identify MC38 tumor cells captured/internalized by CD103+ cDC1s. **b-c**, Percentage of normal MC38 cells captured/internalized by co-cultured CD103+ cDC1s in the presence of different nanoparticles or free PIC at 10 hours (**b**) or 30 hours (**c**) post co-incubation. **d-e**, Percentage of doxorubicin-pretreated MC38 cells captured/internalized by co-cultured CD103+ cDC1s in the presence of different nanoparticles or free PIC at 10 hours (**d**) or 30 hours (**e**) post co-incubation. For **b-e**, n=3 biologically independent samples. For **b-e**, data are presented as mean values ± SEM. Statistical analysis was performed using one-way ANOVA followed by Dunnett test. Source data are provided as a Source Data file.

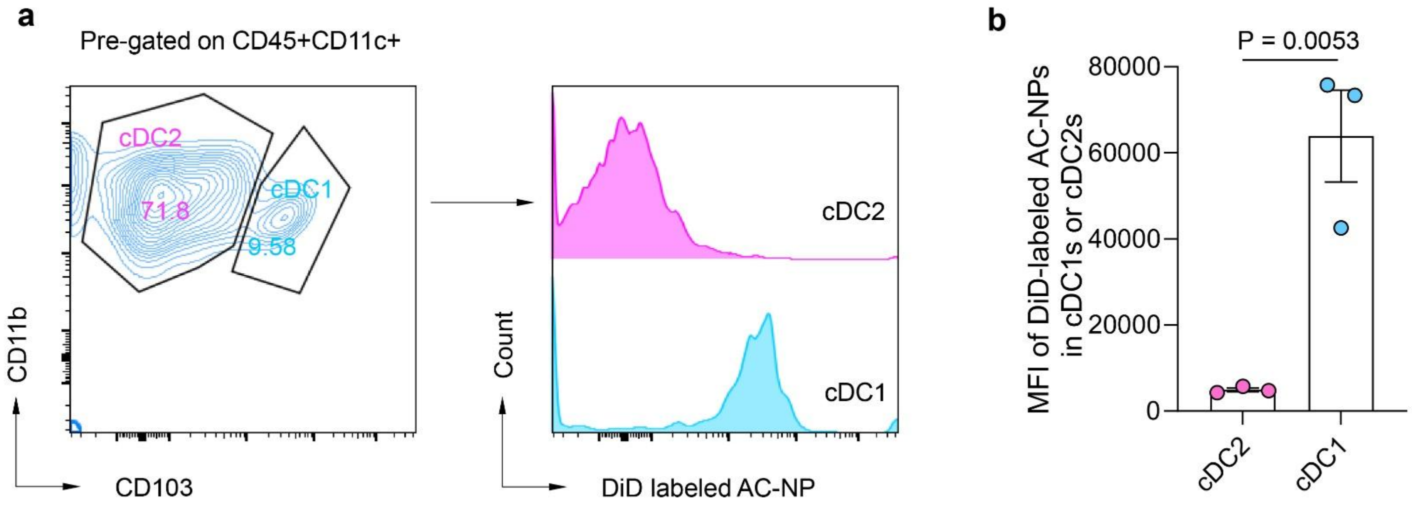

Supplementary Figure 13. AC-NPs showed preferable uptake to cDC1s versus cDC2s when co-incubated with a cell mixture dissociated from a MC38 tumor for 4 hours. AC-NPs were labeled by DiD. After co-incubation, the cell mixture was stained for CD45, CD11c, CD11b, and CD103. **a,** Representative flow cytometry graphs showing the gating strategy for cDC1/cDC2 and relative uptake of AC-NPs by cDC1/cDC2. **b,** MFI of DiD-labeled AC-NPs taken up into cDC1 versus cDC2. n=3 biologically independent samples. Data in (**b**) are presented as mean values ± SEM. Statistical analysis in (**b**) was performed using two-tail student's t test. Source data are provided as a Source Data file.

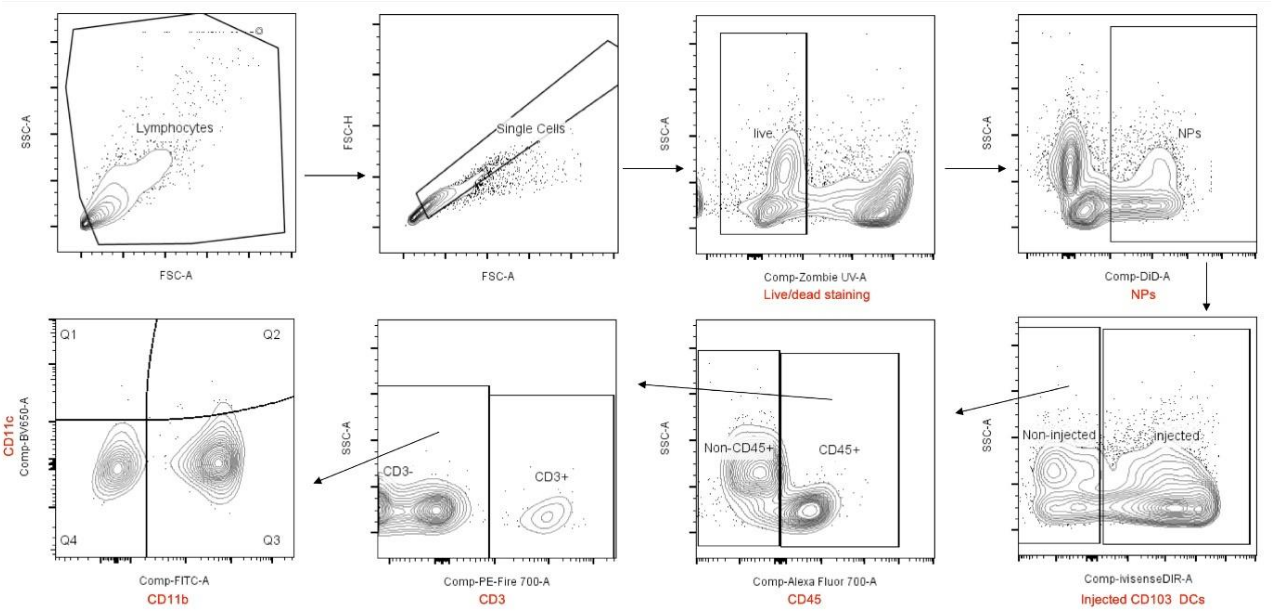

Supplementary Figure 14. Flow cytometry gating strategy to measure the cell-level distribution of AC-NPs 6 hours after intratumoral administration.

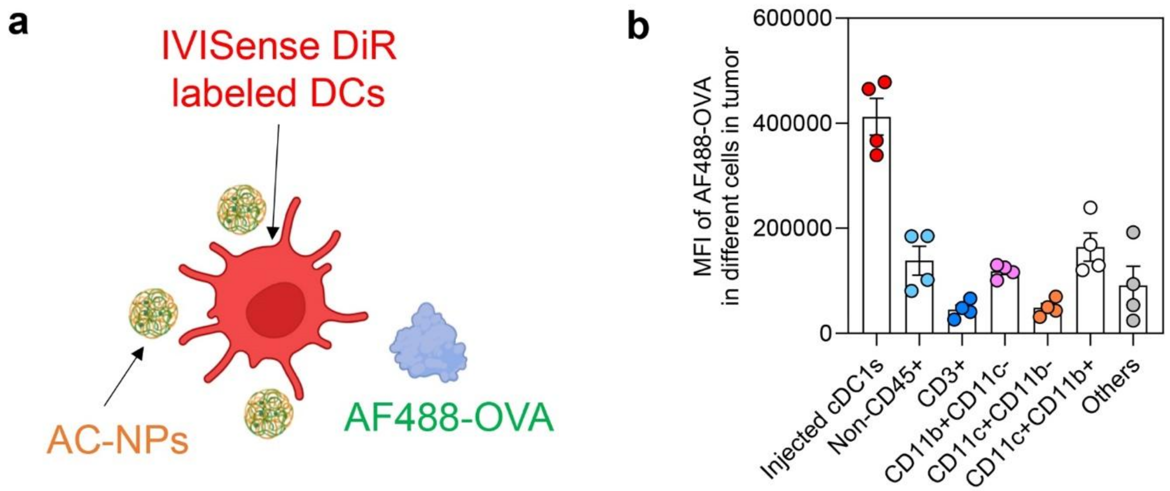

Supplementary Figure 15. Relative amount of model antigen OVA in adoptively injected cDC1s and other tumor-resident cells following intratumoral administration of ACT-DC in the MC38 tumor model. a, Schematic illustrating the experimental design. Created in BioRender. Zhao, Z. (2025) https://BioRender.com/3ow34vf. A model tumor antigen (AF488-OVA) was first intratumorally injected. After 15 minutes, AC-NPs were intratumorally injected, followed by intratumoral injection of IVISense DiR labeled cDC1s another 15 minutes later. Tumors were collected 20 hours post cDC1 injection and processed to quantify the relative amount of AF488-OVA in different cell populations using flow cytometry. **b**, MFI of AF488-OVA in various cells in tumors. n=4 biologically independent mice. Data in (**b**) are presented as mean values ± SEM. Source data are provided as a Source Data file.

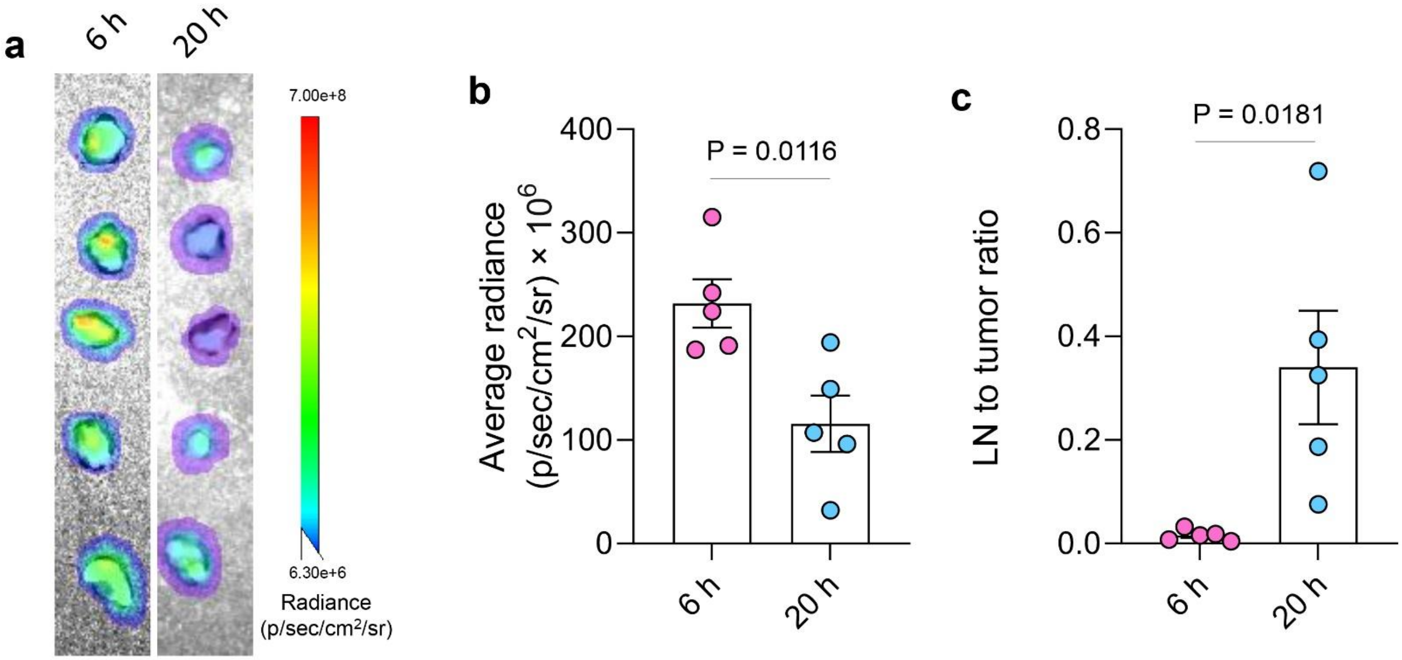

Supplementary Figure 16. Migration of the adoptively transferred CD103+ cDC1s (in ACT-DC) from tumors to tumor draining lymph nodes (tDLNs) upon intratumoral administration. **a**, LagoX images of IVISense DiR labeled CD103+ cDC1s in tumor, 6 or 20 hours after intratumoral administration of ACT-DC. **b**, Relative amount of the adoptively injected CD103+ cDC1s in tumors 6 or 20 hours after intratumoral injection. **c**, The ratio of the number of injected CD103+ cDC1s in tDLNs versus in tumors 6 or 20 hours after intratumoral injection of ACT-DC. n=5 biologically independent mice per group. Data in (**b-c**) are presented as mean values ± SEM. Statistical analysis was performed using two-tail student's t test. Source data are provided as a Source Data file.

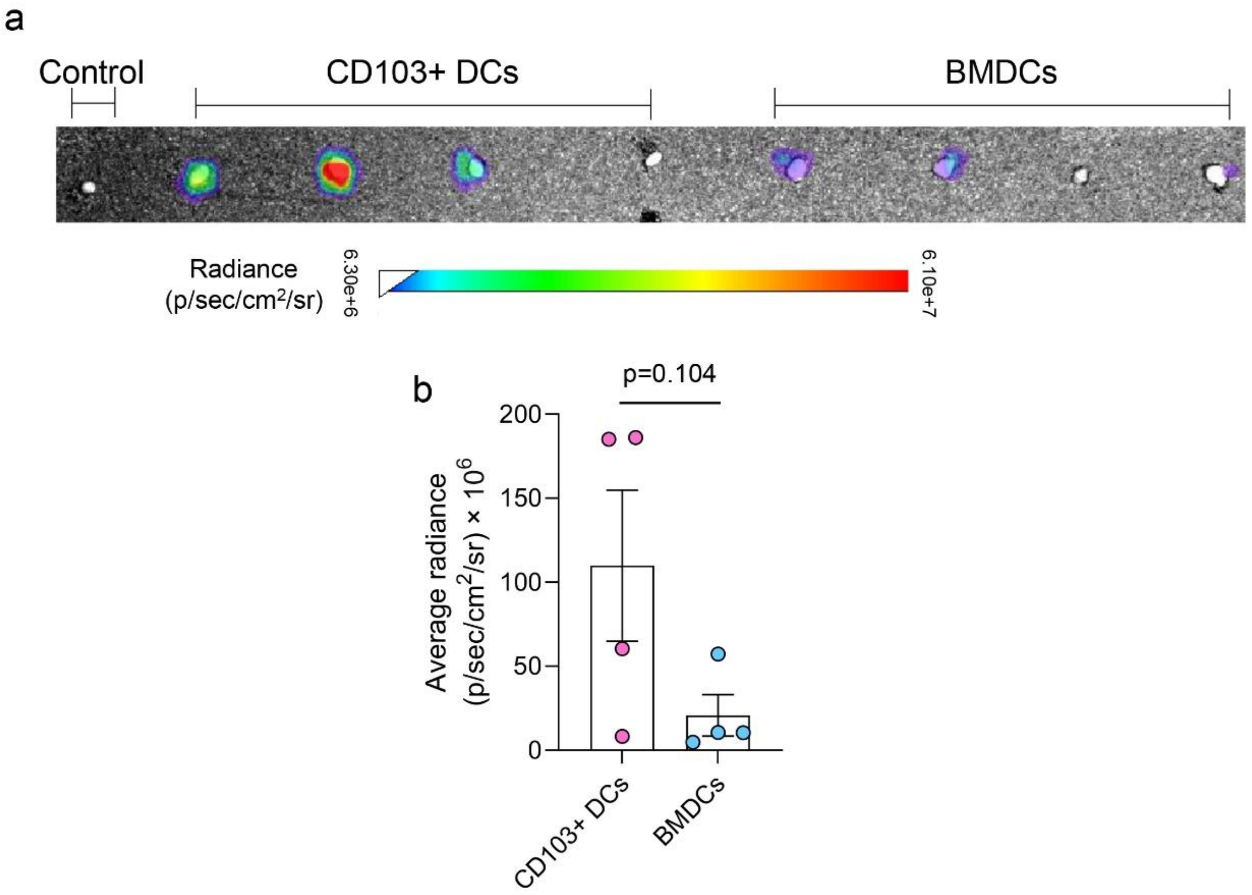

Supplementary Figure 17. Comparison of the trafficking of CD103+ cDC1s vs bone marrow derived DCs (BMDCs) to tDLNs. CD103+ cDC1s and BMDCs were stained with IVISense DiR. cDC1s+AC-NPs or BMDCs+AC-NPs were intratumorally injected, and the relative number of injected DCs migrated to tDLNs at 20 h were measured by LagoX. **a**, LagoX images of tDLNs. **b**, Relative amount of the injected CD103+ cDC1s or BMDCs migrated to tDLNs 20 hours after intratumoral injection. n=4 biologically independent mice per group. For **b**, data are presented as mean values ± SEM. Statistical analysis was performed using two-tailed student's t test. Source data are provided as a Source Data file.

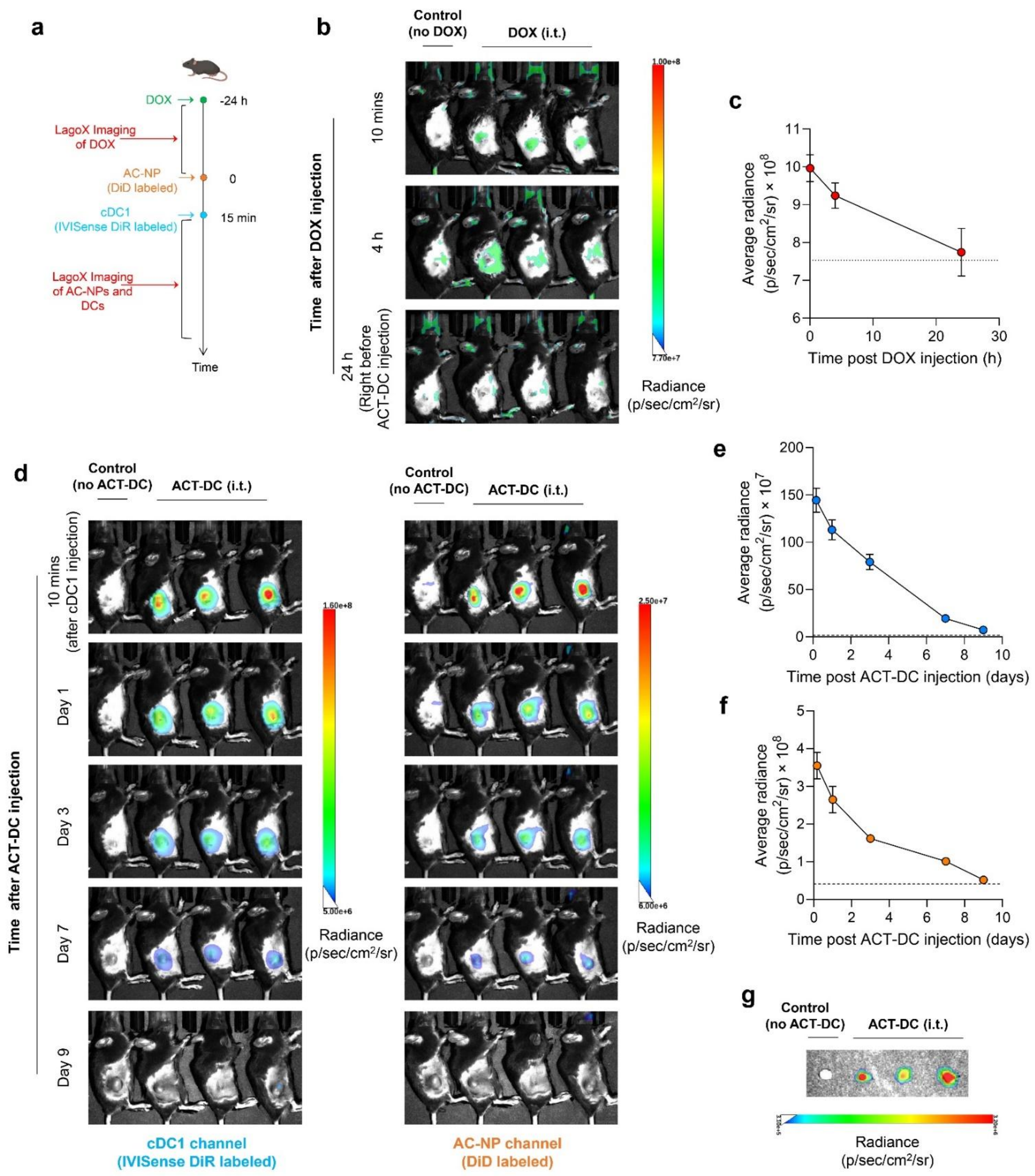

Supplementary Figure 18. Retention kinetics of doxorubicin (DOX) and ACT-DC (AC-NPs and cDC1s) following sequential intratumoral injection. a, Schematic showing experimental design. Created in BioRender. Zhao, Z. (2025) https://BioRender.com/3ow34vf. **b**, LagoX images of DOX signals in MC38 tumor mice at different time points. **c**, Fluorescence intensity of DOX at the tumor site following intratumoral injection. Dashed line indicates the background value from the control mice at 24 h. **d**, LagoX images of cDC1 and AC-NP signals at different time points. **e**, Fluorescence intensity of cDC1 (labeled by IVISense DiR) at the tumor site. Dashed line indicates the background value from the control mice on day 9. **f**, Fluorescence intensity of AC-NP (labeled by DiD) at the tumor site. Dashed line indicates the background value from the control mice on day 9. **g**, LagoX images of tDLNs collected on day 9. n=4 biologically independent mice. Data in (**c, e, f**) are presented as mean values ± SEM. Source data are provided as a Source Data file.

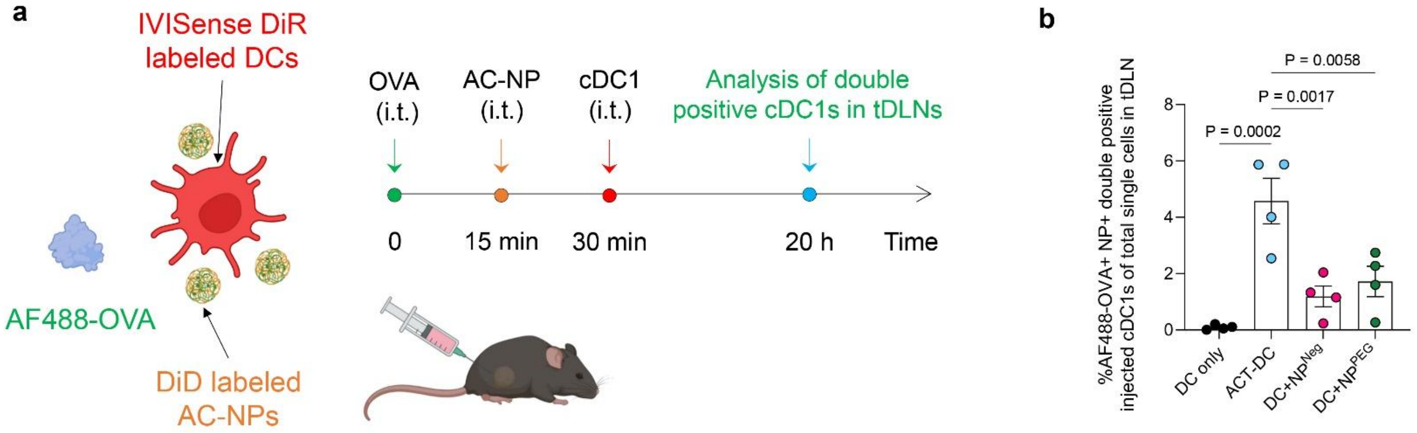

Supplementary Figure 19. ACT-DC enhanced the accumulation of model tumor antigen and NP double positive cDC1s in tDLNs in the MC38 tumor model. a, Schematic depicting the experimental design to measure the number of injected cDC1s that are double positive for model tumor antigen and NPs. Created in BioRender. Zhao, Z. (2025) https://BioRender.com/3ow34vf. **b**, Relative number of double positive injected cDC1s in tDLNs. For **b**, data are presented as mean values ± SEM. Statistical analysis was performed using one-way ANOVA followed by Dunnett test. n=4 biologically independent animals per group. Source data are provided as a Source Data file.

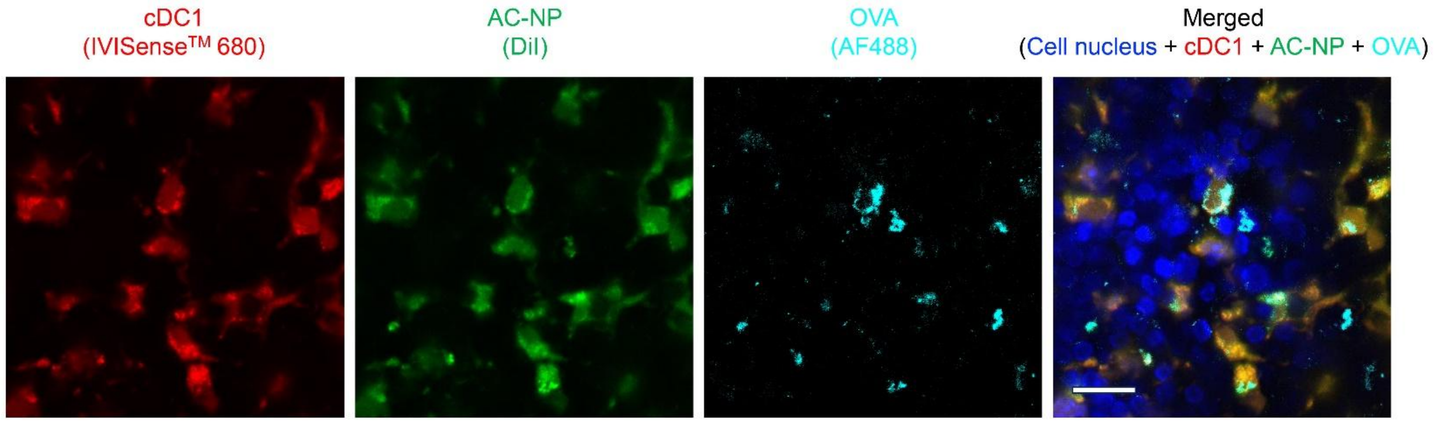

Supplementary Figure 20. CLSM images of tDLN showing the presence of AC-NPs and the model antigen AF488-OVA within the injected cDC1s that migrated to tDLNs. This study was conducted in the MC38 tumor model. OVA (AF488 labeled), AC-NP (DiI labeled), and cDC1s (IVISense 680 labeled) were sequentially intratumorally injected (15 mins apart). tDLN was collected 20 hours after cDC1 injection. Representative of two independent experiments with similar results. Scale bars: 20 μm.

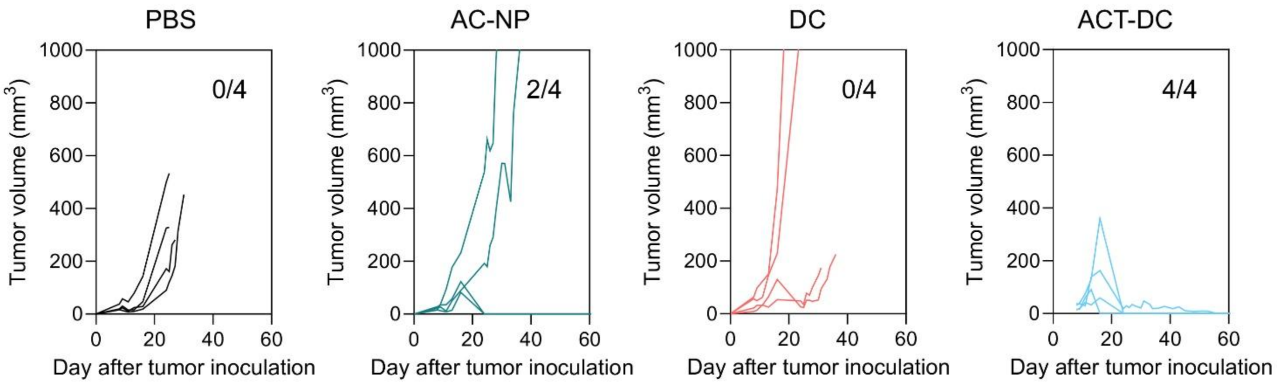

Supplementary Figure 21. Tumor growth curve of individual mice in the early-stage MC38 tumor model shown in Fig. 3a-c. n=4 biologically independent mice per group. Source data are provided as a Source Data file.

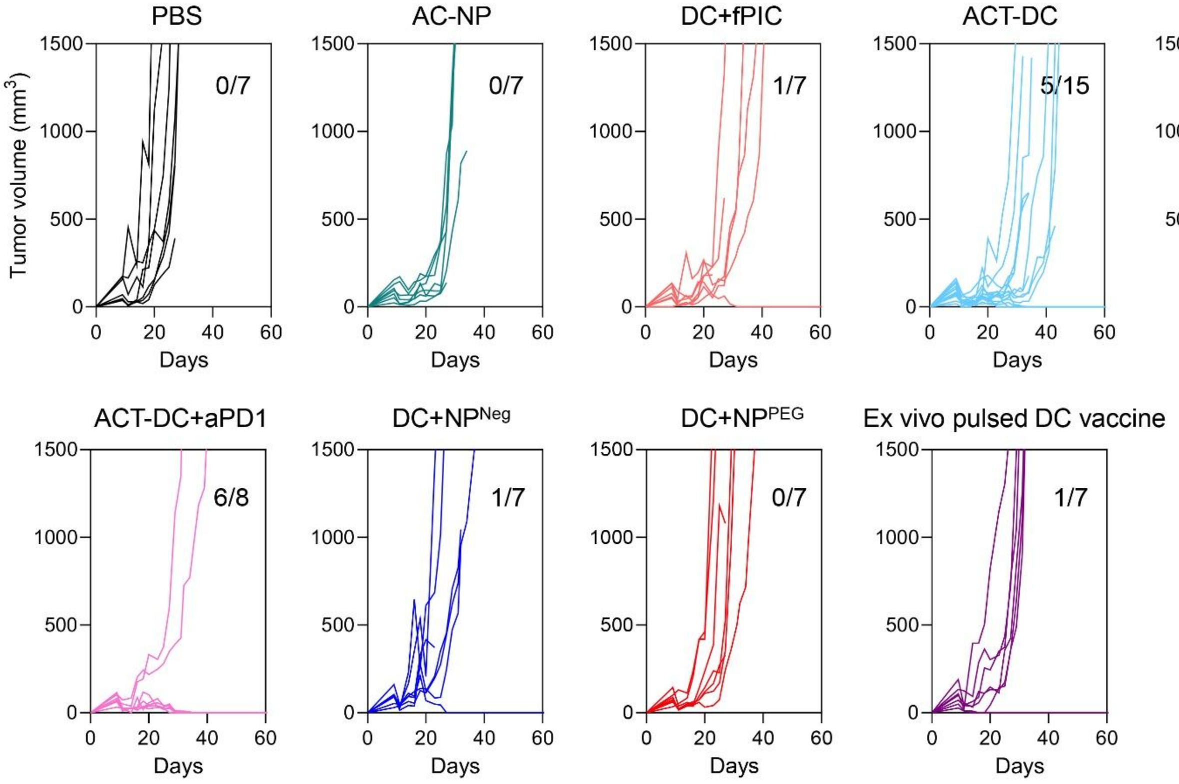

Supplementary Figure 22. Tumor growth curve of individual mice in the large-established MC38 tumor model shown in Fig. 3e-f. n=15 for ACT-DC, n=8 for ACT-DC+aPD1, n=7 for the other groups, biologically independent animals. Source data are provided as a Source Data file.

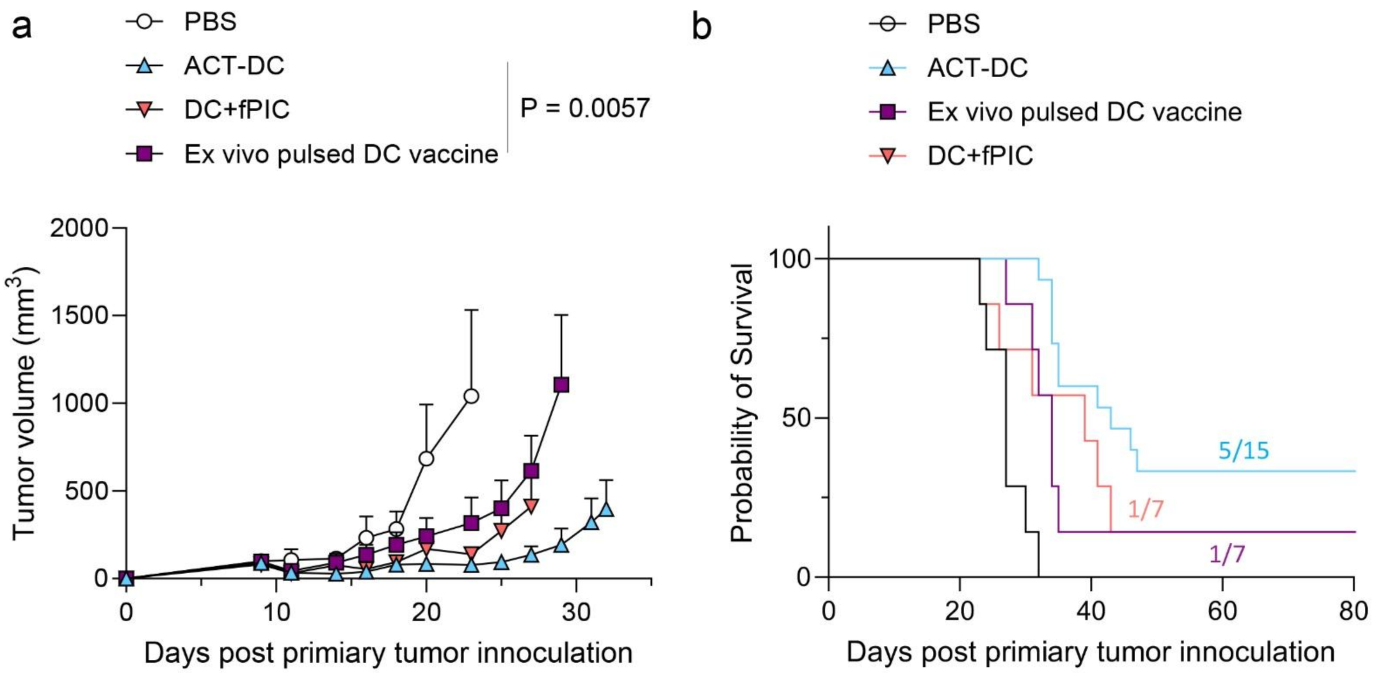

Supplementary Figure 23. Comparison of the therapeutic efficacy of ACT-DC to that of conventional *ex vivo* tumor lysate pulsed DC vaccine or DC + free PIC in the large-established MC38 tumor model. a, Tumor growth curve. **b,** Survival curve. n=15 for ACT-DC, n=7 for the other groups, biologically independent animals. Data in (**a**) are presented as mean values ± SEM. Statistical analysis in (**a**) was performed using two-way ANOVA followed by Dunnett test. Source data are provided as a Source Data file.

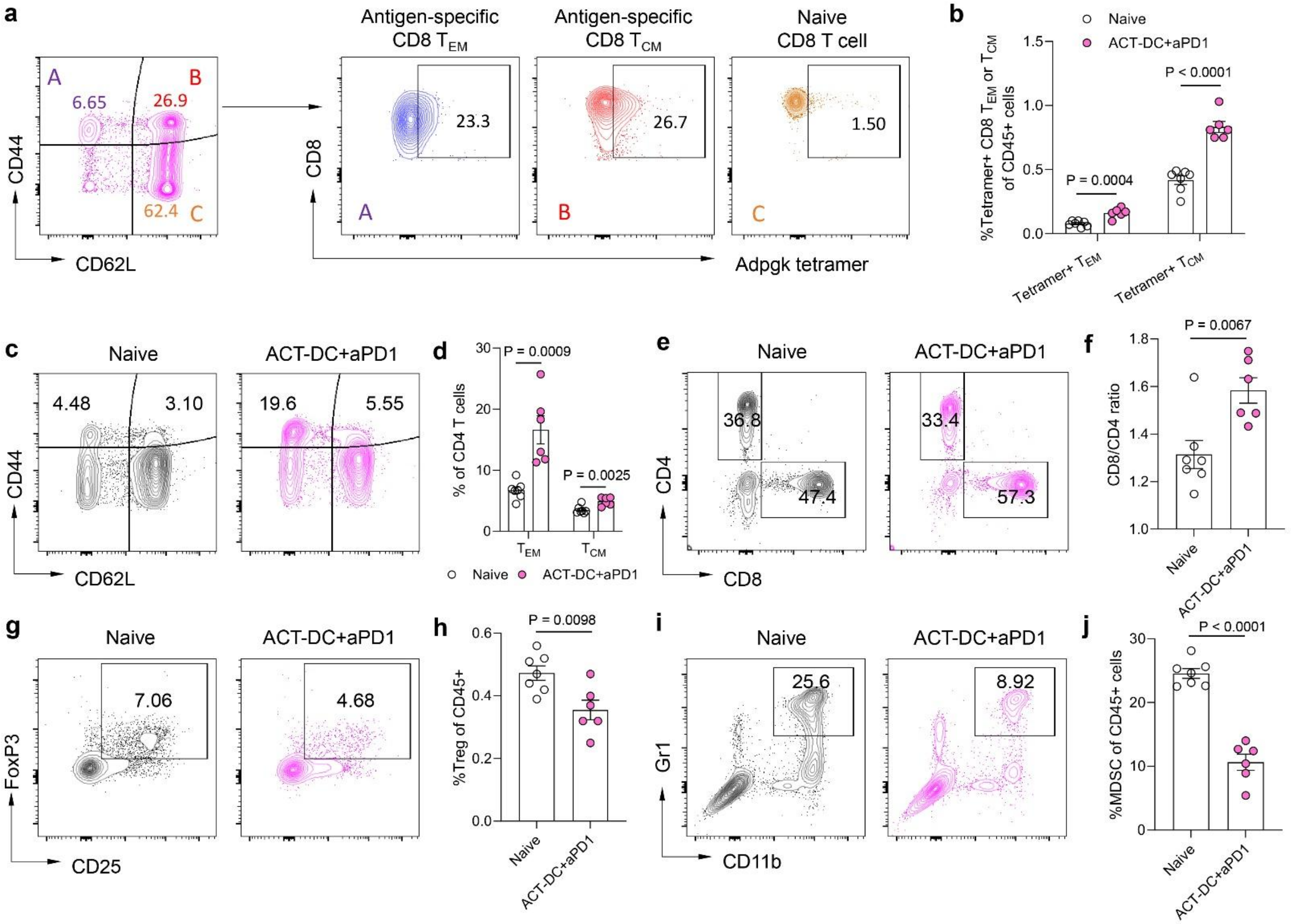

Supplementary Figure 24. Immune cell profiles in the blood of mice 15 days after the 2nd rechallenge in the large-established MC38 tumor model. a-b, Representing flow gating strategy (**a**) and quantification (**b**) of Adpgk tetramer positive effector memory and central memory CD8 T cells in the blood. **c-d,** Representative flow plot and quantification of memory CD4 T cells in the blood. **e-f**, Representative flow plot and the relative ratio of CD8 to CD4 T cells in the blood. **g-h**, Representative flow plot and quantification of Tregs in the blood. **i-j**, Representative flow plot and quantification of myeloid derived suppressor cells (MDSCs) in the blood. n=7 for Naïve, n=6 for ACT-DC+aPD1, biologically independent animals. Data in (**b, d, f, h, j**) are presented as mean values ± SEM. Statistical analysis in (**b, d, f, h, j**) was performed using two-tail student's t test. Source data are provided as a Source Data file.

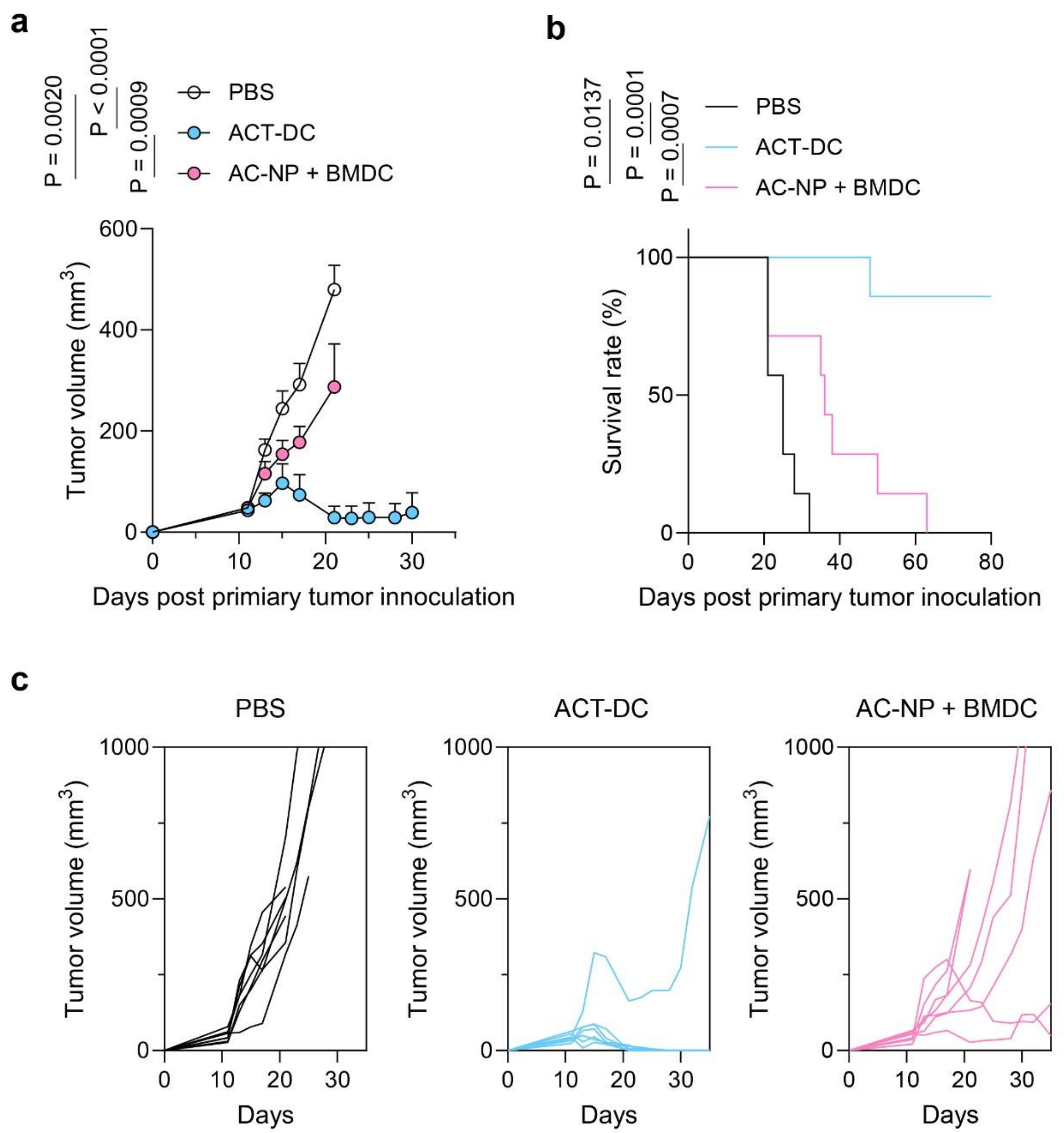

Supplementary Figure 25. Comparison of the therapeutic efficacy of ACT-DC versus BMDC + AC-NP in the MC38 tumor model. **a**, Tumor growth curve. **b**, Survival curve. **c**, Tumor growth curve of individual mice. For **a-c**, n=7 biologically independent mice per group. Data in (**a**) are presented as mean values ± SEM. For **a**, statistical analysis was performed using two-way ANOVA followed by Dunnett test. For **b**, statistical analysis was performed using two-sided Mantel–Cox tests. Source data are provided as a Source Data file.

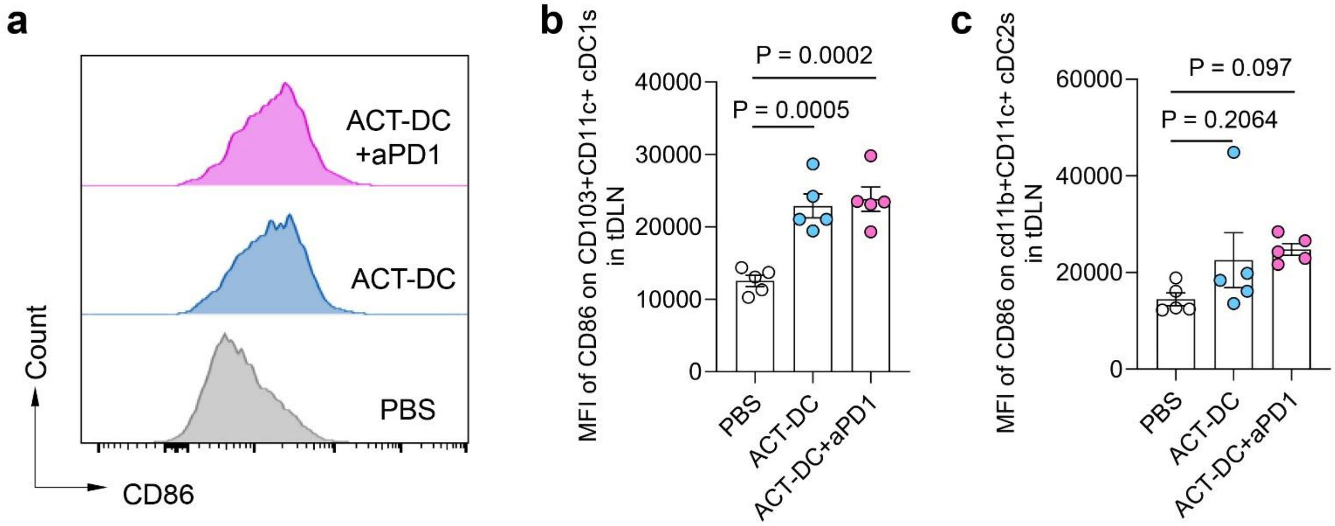

Supplementary Figure 26. Activation status of cDC1s and cDC2s in the tDLNs after two doses of different therapies. a, Representative flow plot showing the expression of CD86 on CD103+CD11c+ cDC1s in the tDLNs. **b,** MFI of CD86 on CD103+CD11c+ cDC1s in tDLN. **c**, MFI of CD86 on CD11b+CD11c+ cDC2s in tDLNs. n=5 biologically independent mice per group. Data in (**b-c**) are presented as mean values ± SEM. Statistical analysis was performed using one-way ANOVA followed by Dunnett test. Source data are provided as a Source Data file.

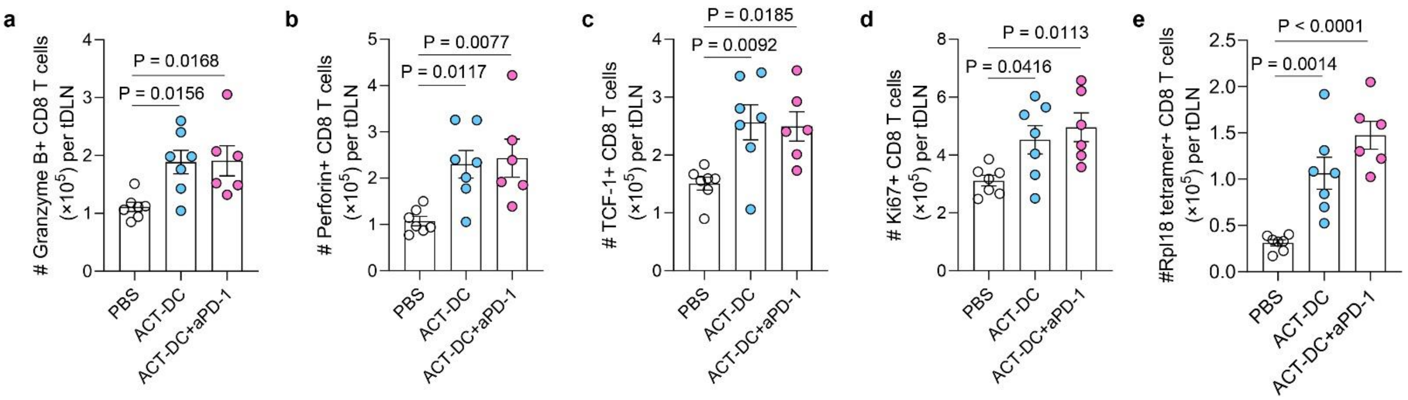

Supplementary Figure 27. Effector, proliferating, and antigen-specific CD8 T cells in the tDLNs after two doses of ACT-DC therapies in the MC38 tumor model. a, Granzyme B+ CD8 T cells. **b,** Perforin+ CD8 T cells. **c**, TCF-1+ CD8 T cells. **d**, Ki67+ CD8 T cells. **e**, Rpl18 tetramer specific CD8 T cells. n=7 for PBS and ACT-DC, n=6 for ACT-DC+aPD1, biologically indepent mice. Statistical analysis was performed using one-way ANOVA followed by Dunnett test. Data in this figure were collected from a separate study according to the same experimental schedule shown in Fig. 4a. Data in (**a-e**) are presented as mean values ± SEM. Source data are provided as a Source Data file.

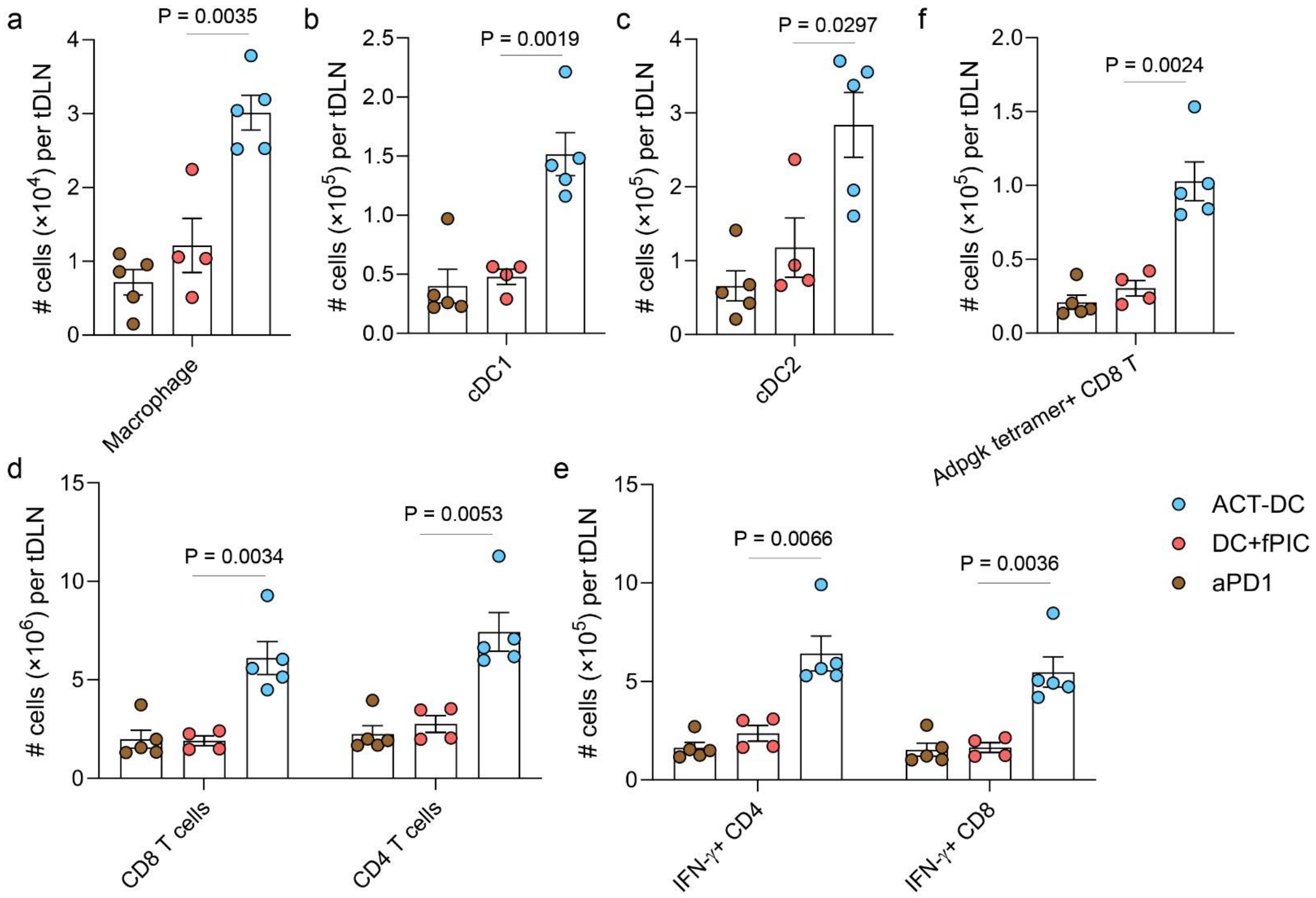

Supplementary Figure 28. Immune cell profiles in the tDLNs after two doses of different therapies. a, Macrophages. **b,** cDC1. **c**, cDC2. **d**, CD8 and CD4 T cells. **e**, IFN-γ-expressing CD4 and CD8 T cells. **f**, Adpgk tetramer positive CD8 T cells. n=4 for DC+fPIC, n=5 for the other groups, biologically independent mice. Data in (**a-f**) are presented as mean values ± SEM. Statistical analysis was performed using two-tail student's t test. Source data are provided as a Source Data file.

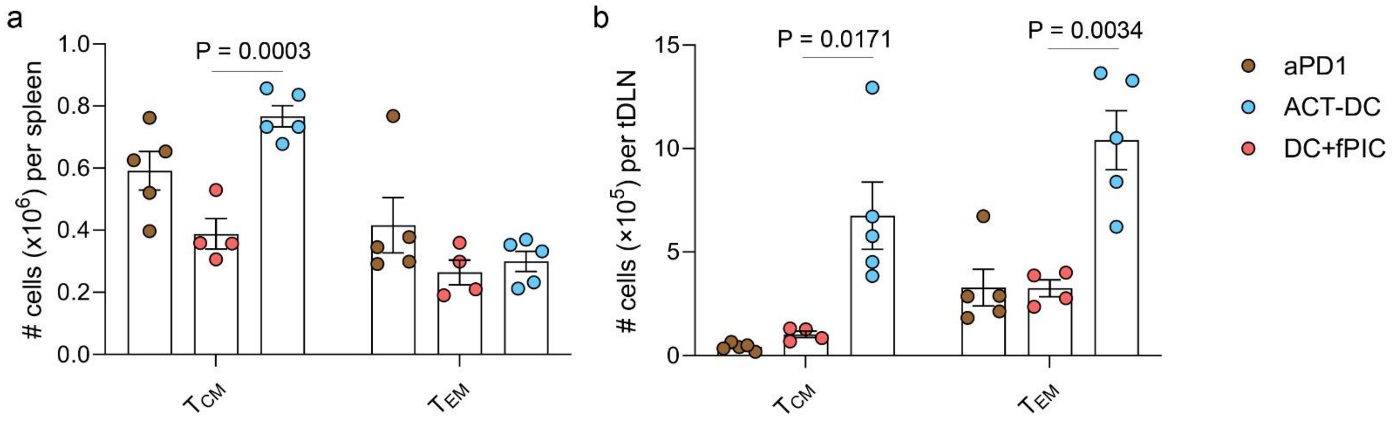

Supplementary Figure 29. Memory CD8 T cells in the spleen and tDLNs after two doses of different therapies. The number of central memory (CD44+CD62L+) and effector memory (CD44+CD62L-) CD8 T cells in the spleen (**a**) and tDLNs (**b**) were shown. n=4 for DC+fPIC, n=5 for the other groups, biologically independent mice. Data in (**a-b**) are presented as mean values ± SEM. Statistical analysis was performed using two-tail student's t test. Source data are provided as a Source Data file.

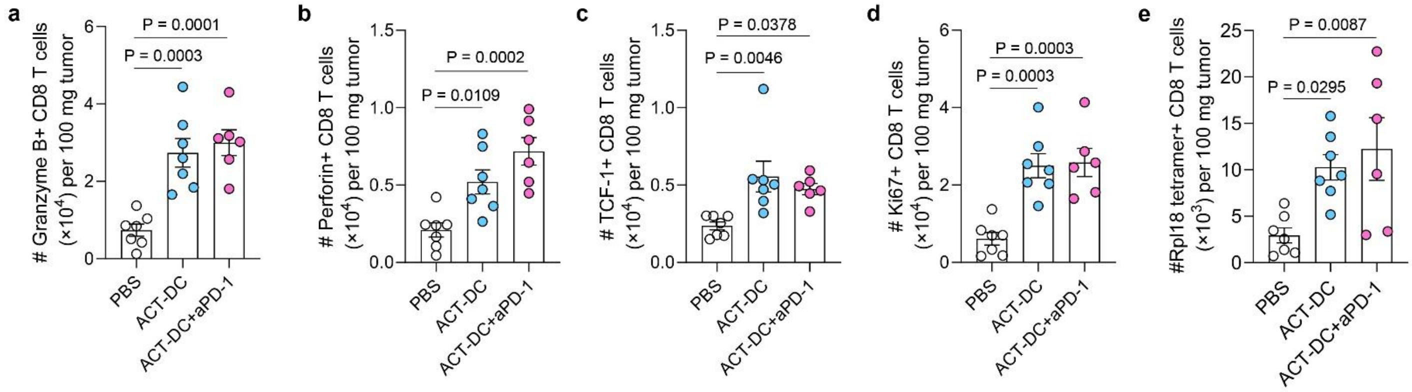

Supplementary Figure 30. Effector, proliferating, and antigen-specific CD8 T cells in the tumor after two doses of ACT-DC therapies in the MC38 tumor model. a, Granzyme B+ CD8 T cells. **b,** Perforin+ CD8 T cells. **c**, TCF-1+ CD8 T cells. **d**, Ki67+ CD8 T cells. **e**, RPL18 tetramer positive CD8 T cells. n=7 for PBS and ACT-DC, n=6 for ACT-DC+aPD1, biologically indepent mice. Statistical analysis was performed using one-way ANOVA followed by Dunnett test. Data in this figure were collected from a separate study according to the same experimental schedule shown in Fig. 5a. Data in (**a-e**) are presented as mean values ± SEM. Source data are provided as a Source Data file.

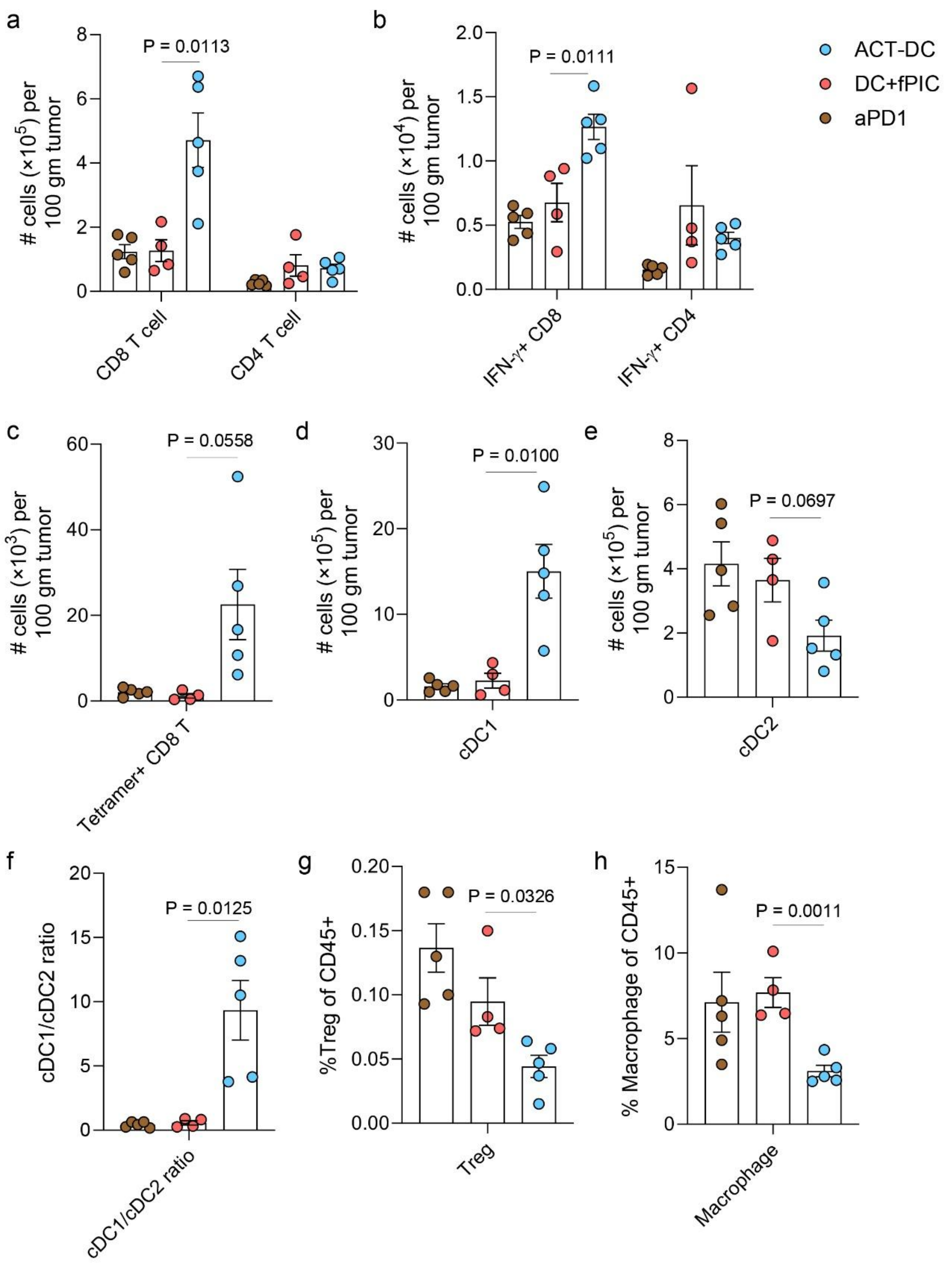

Supplementary Figure 31. Immune cell profiles in the tumor after two doses of different therapies. a, CD4 and CD8 T cells. **b,** IFN-γ-expressing CD4 and CD8 T cells. **c**, Adpgk tetramer positive CD8 T cells. **d**, cDC1. **e**, cDC2. **f**, Relative ratio of cDC1 to cDC2. **g**, Tregs. **h**, macrophages. n=4 for DC+fPIC, n=5 for the other groups, biologically independent mice. Data in (**a-h**) are presented as mean values ± SEM. Statistical analysis was performed using two-tail student's t test. Source data are provided as a Source Data file.

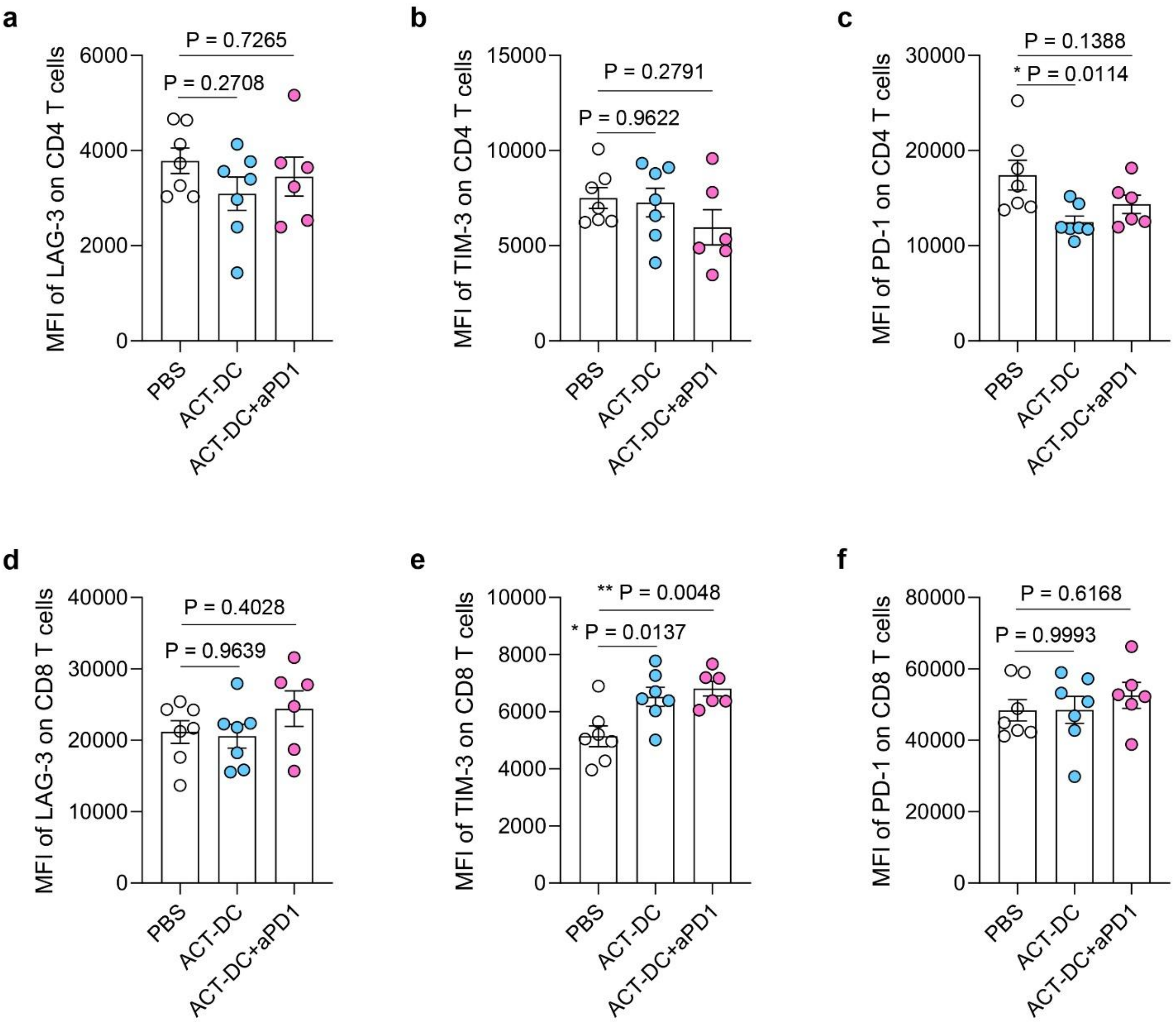

Supplementary Figure 32. Expression of exhaustion markers on intratumoral CD4 and CD8 T cells after two doses of ACT-DC therapy in the MC38 tumor model. a-c, Relative expression levels of LAG-3 (**a**), TIM-3 (**b**), and PD-1 (**c**) on CD4 T cells in tumors. **d-f,** Relative expression levels of LAG-3 (**d**), TIM-3 (**e**), and PD-1 (**f**) on CD8 T cells in tumors. Statistical analysis was performed using one-way ANOVA followed by Dunnett test. Data in this figure were collected from a separate study according to the same experimental schedule shown in Fig. 5a. Fig. S27, Fig. S30, and Fig. 32 were from the same study. n=7 for PBS and ACT-DC, n=6 for ACT-DC+aPD1, biologically indepent mice. Data in (**a-f**) are presented as mean values ± SEM. Source data are provided as a Source Data file.

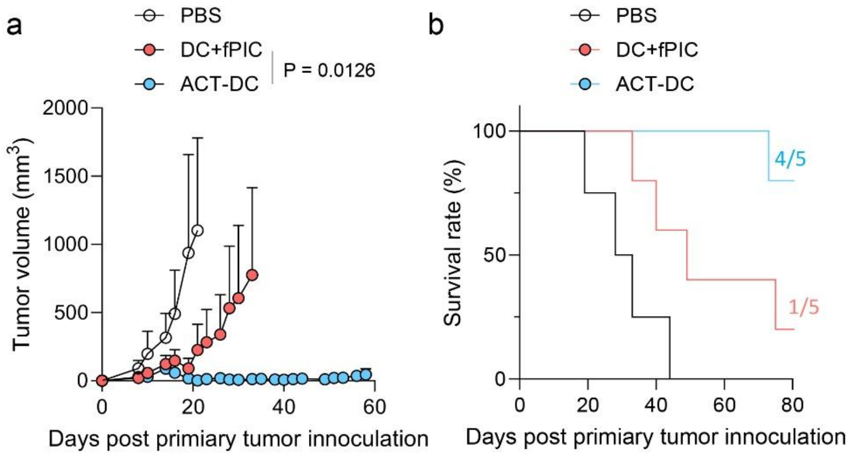

Supplementary Figure 33. Comparison of the therapeutic efficacy of ACT-DC to DC+free PIC in the B16F10 tumor model. a, Tumor growth curve. **b,** Survival curve. n=4 for PBS, n=5 for the other groups, biologically independent mice. Data in (**a**) are presented as mean values ± SEM. Statistical analysis in (**a**) was performed using two-way ANOVA followed by Dunnett test. Source data are provided as a Source Data file.

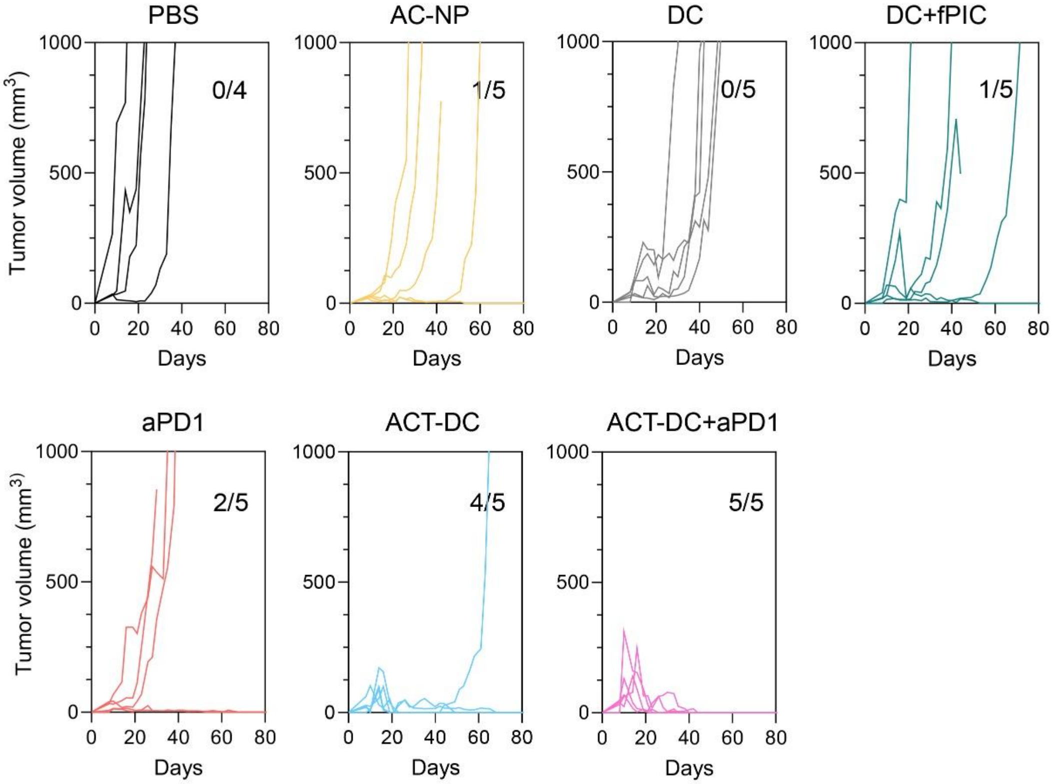

Supplementary Figure 34. Tumor growth curve of individual mice in the B16F10 tumor model shown in Fig. 6b-c. n=4 for PBS, n=5 for the other groups, biologically independent mice. Source data are provided as a Source Data file.

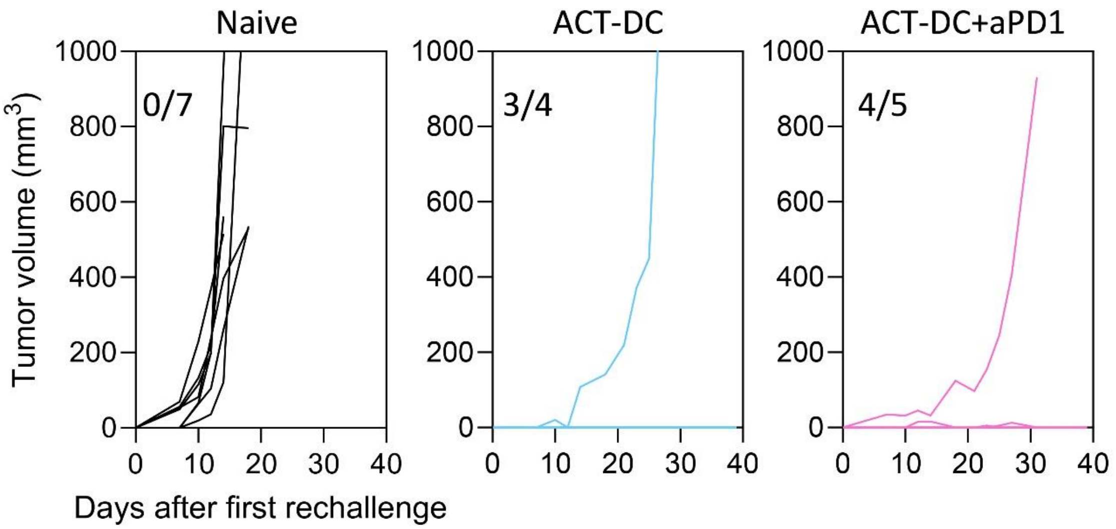

Supplementary Figure 35. Efficacy of ACT-DC in controlling the 1st tumor rechallenge (s.c.) in the B16F10 model. Tumor growth curve of individual mice shown in Fig. 6d-e. n=7 for Naïve, n=4 for ACT-DC, n=5 for ACT-DC+aPD1, biologically independent mice. Source data are provided as a Source Data file.

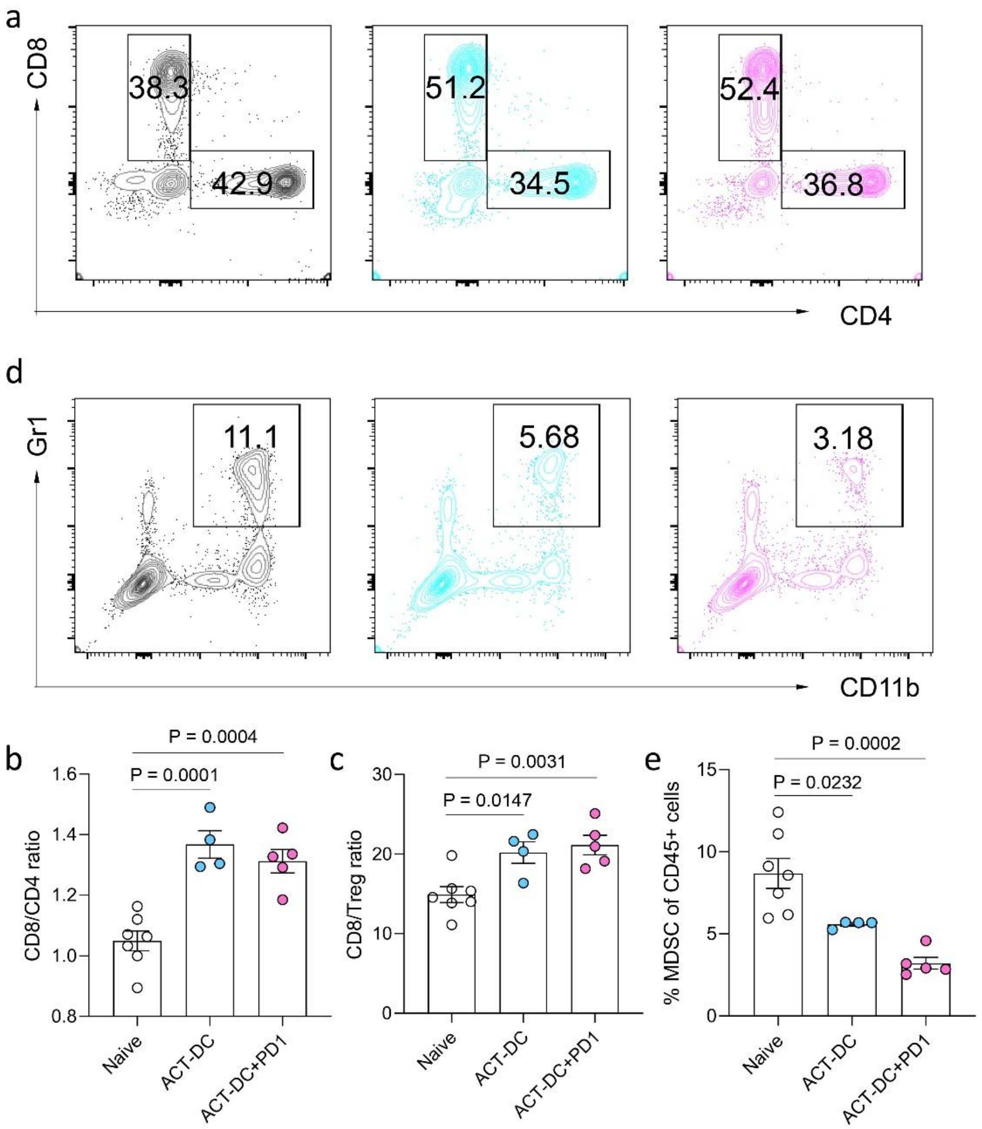

Supplementary Figure 36. Immune cell profiles in the blood of mice 14 days after the 1st tumor rechallenge in the B16F10 tumor model. a-b, Representative flow plot and the relative ratio of CD8 to CD4 T cells in the blood. **c**, CD8 to Treg ratio in the blood, **d-e**, Representative flow plot and quantification of MDSCs in the blood. n=7 for Naïve, n=4 for ACT-DC, n=5 for ACT-DC+aPD1, biologically independent mice. Data in (**b, c, e**) are presented as mean values ± SEM. Statistical analysis in (**b, c, e**) was performed using one-way ANOVA followed by Dunnett test. Source data are provided as a Source Data file.

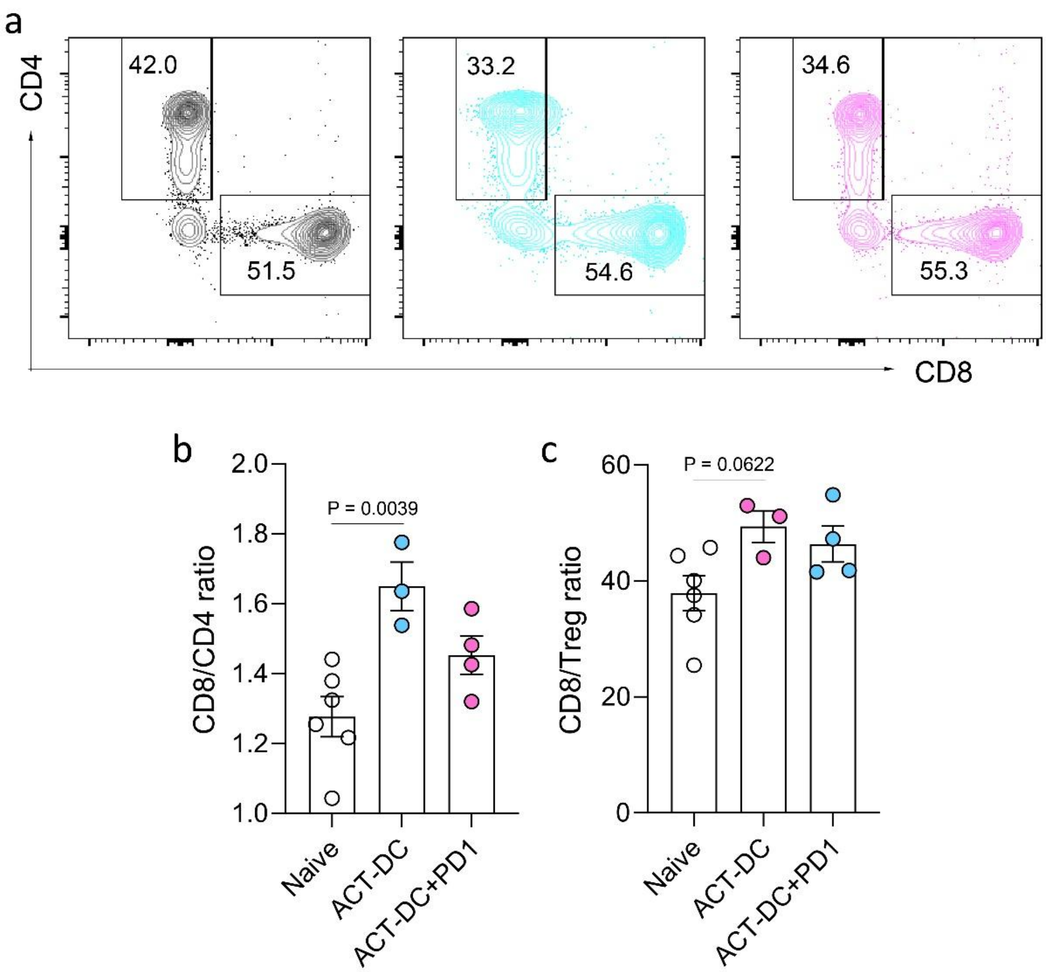

Supplementary Figure 37. Immune cell profiles in the blood of mice 15 days after the 2nd tumor rechallenge (i.v.) in the B16F10 tumor model. a-b, Representative flow plot and the relative ratio of CD8 to CD4 T cells in the blood. **c**, CD8 to Treg ratio in the blood. n=6 for Naïve, n=3 for ACT-DC, n=4 for ACT-DC+aPD1, biologically independent mice. Data in (**b-c**) are presented as mean values ± SEM. Statistical analysis in (**b, c**) was performed using one-way ANOVA followed by Dunnett test. Source data are provided as a Source Data file.

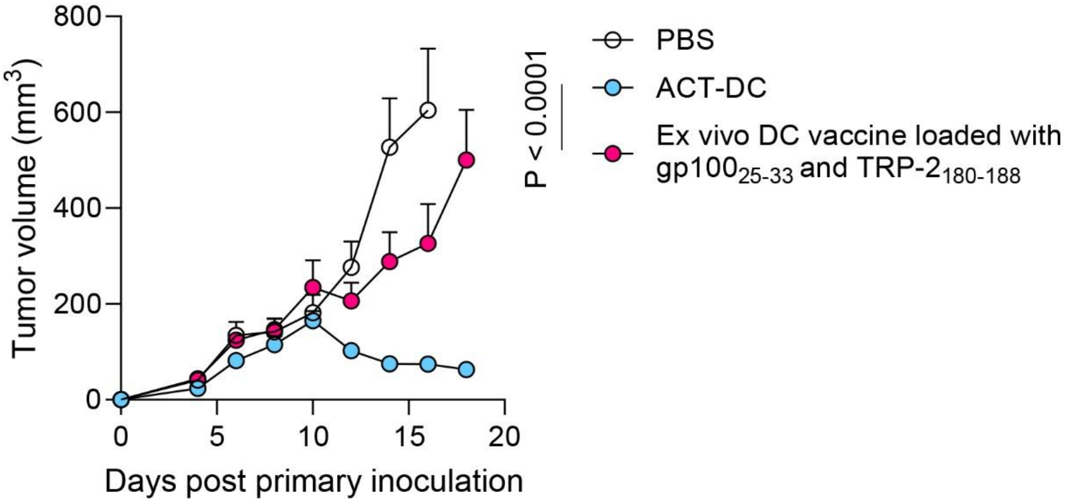

Supplementary Figure 38. Comparison of the therapeutic efficacy of ACT-DC to *ex vivo* DC vaccine loaded with gp100$_{25\text{-}33}$ and TRP-2$_{180\text{-}188}$ in the B16F10 tumor model. Tumor growth curve is shown (n=7 independent animals per group). Data are expressed as mean values ± SEM. Statistical analysis was performed using two-way ANOVA followed by Dunnett test. Source data are provided as a Source Data file.

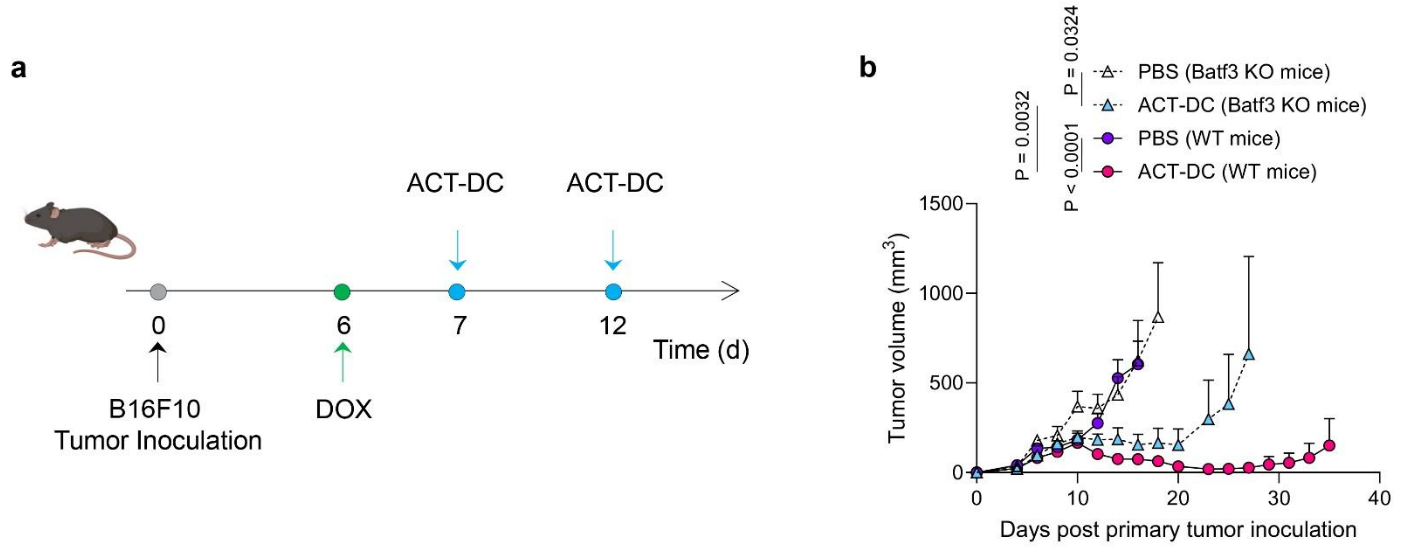

Supplementary Figure 39. Effect of endogenous cDC1s on the therapeutic efficacy of ACT-DC in the B16F10 tumor model. a, Schedule of the study. Created in BioRender. Zhao, Z. (2025) https://BioRender.com/3ow34vf. **b,** Tumor growth curve. n=7 biologically independent mice per group for wild-type mice; for Batf3$^{-/-}$ mice, n=4 for PBS, n=6 for ACT-DC, biologically independent mice. Data are expressed as mean ± SEM. Statistical analysis was performed using two-way ANOVA followed by Dunnett test. Source data are provided as a Source Data file.

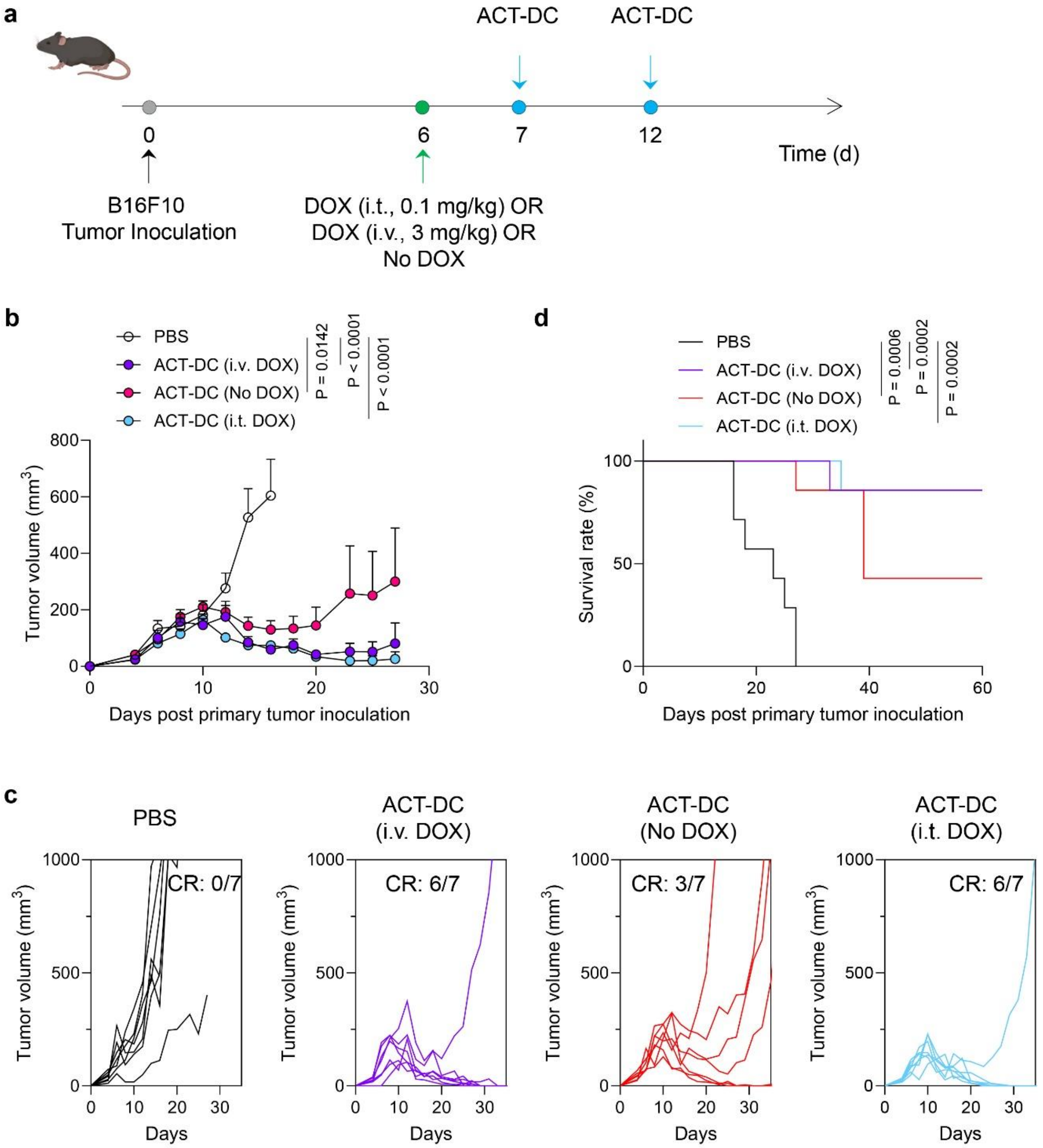

Supplementary Figure 40. Impact of doxorubicin pre-treatment on the therapeutic efficacy of ACT-DC in the B16F10 melanoma model. a, Schematic showing the treatment schedule. Created in BioRender. Zhao, Z. (2025) https://BioRender.com/3ow34vf. **b**, Overall tumor growth curve. **c**, Individual tumor growth curve. **d**, Survival curve. n=7 biologically independent mice per group. Data in (**b**) are expressed as mean ± SEM. Statistical analysis in (**b**) was performed using two-way ANOVA followed by Dunnett test. Statistical analysis in (**d**) was performed using two-sided Mantel-Cox tests. Source data are provided as a Source Data file.

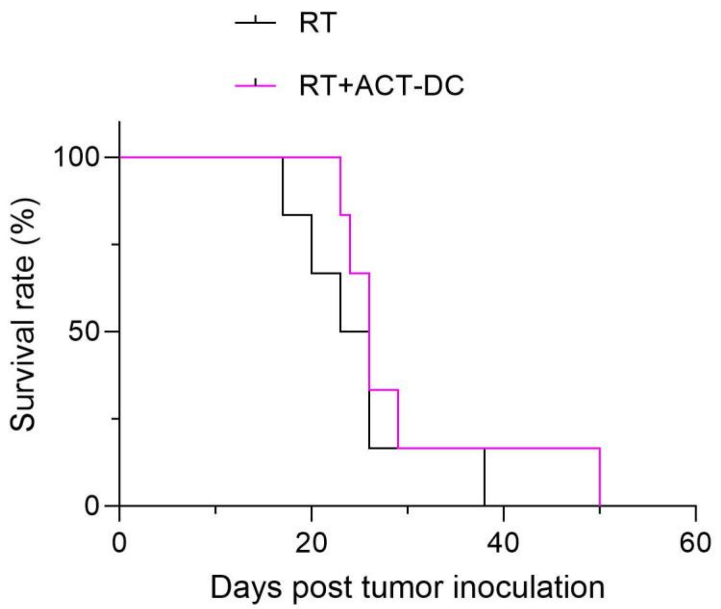

Supplementary Figure 41. Survival of mice receiving radiation treatment (RT) alone or RT combined with ACT-DC in CT-2A glioma model. Mice were treated according to the schedule in Fig. 7a. This is a separate study compared to Fig. 7b. n=6 biologically independent mice per group. Source data are provided as a Source Data file.

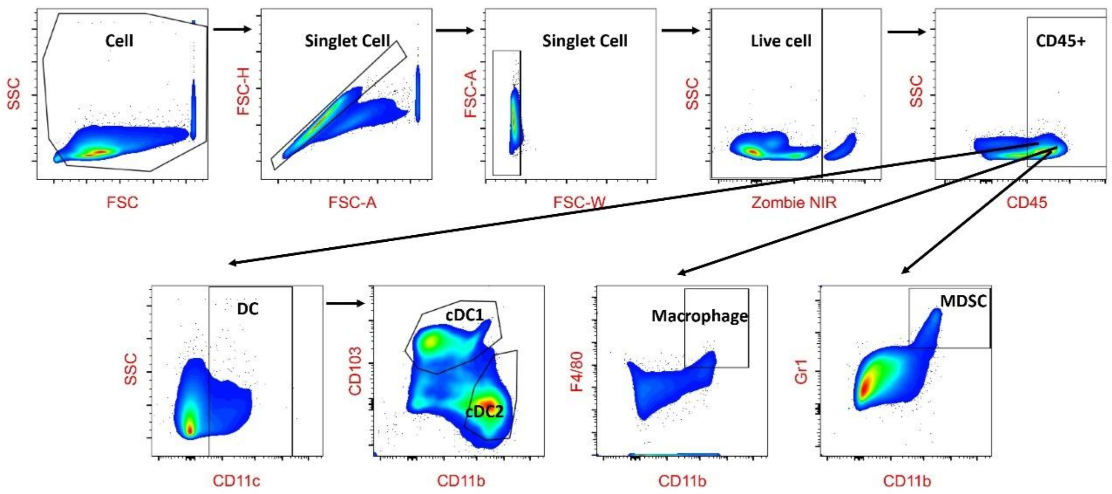

Supplementary Figure 42. Representative flow gating strategies for DCs, macrophages, and MDSCs (Figs. 4b-d, 5g-i, 5l and Supplementary Figs. 28a-c, 31d-h, 36e).

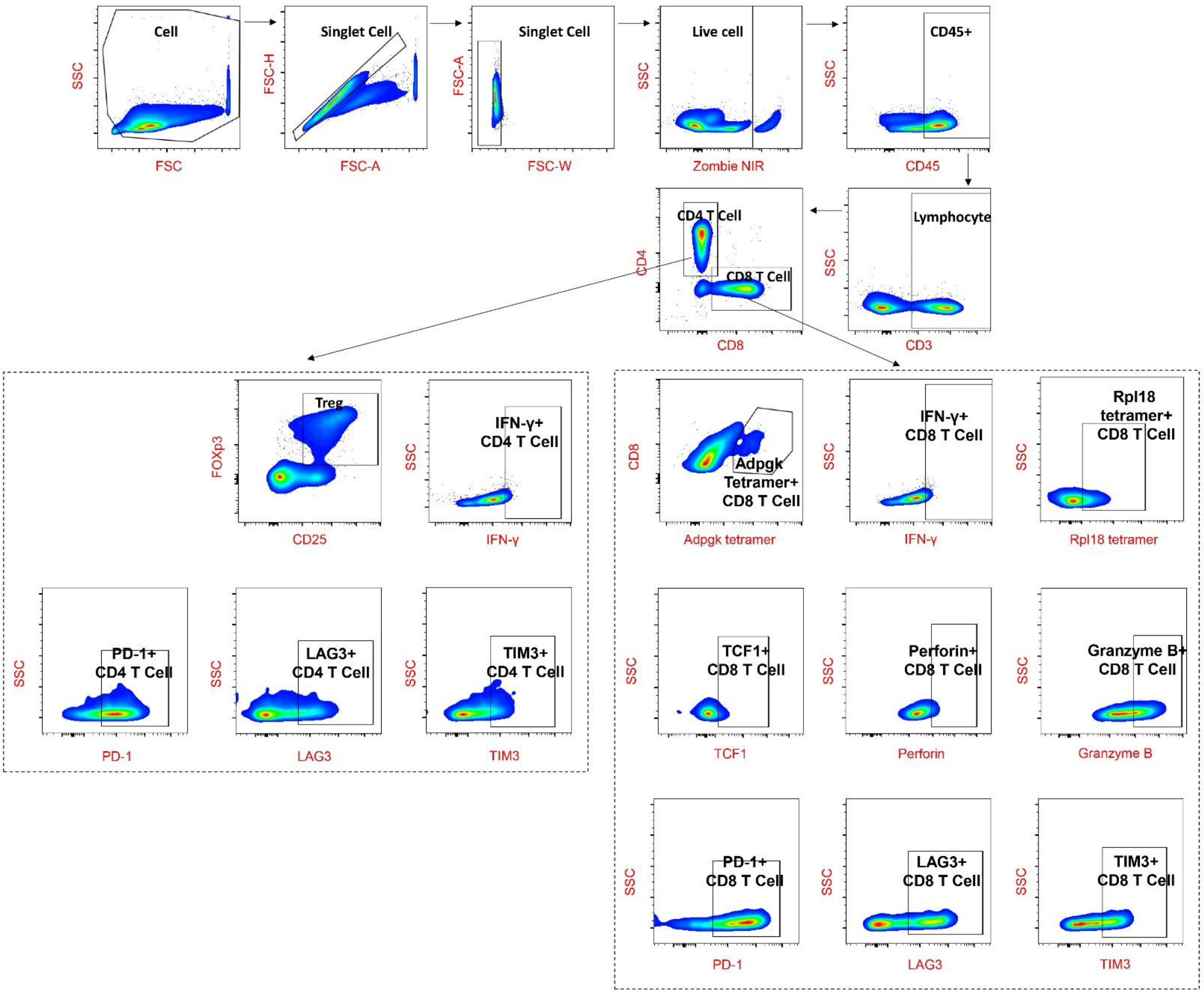

Supplementary Figure 43. Representative flow gating strategies for T cells, Th1 cells, Tregs, effector CD8 T cells, and exhaustion marker expression on T cells (Figs. 4e-g, 5b-e, 5k and Supplementary Figs. 27, 28d-f, 30, 31a-c, 32, 36a-c, 37).

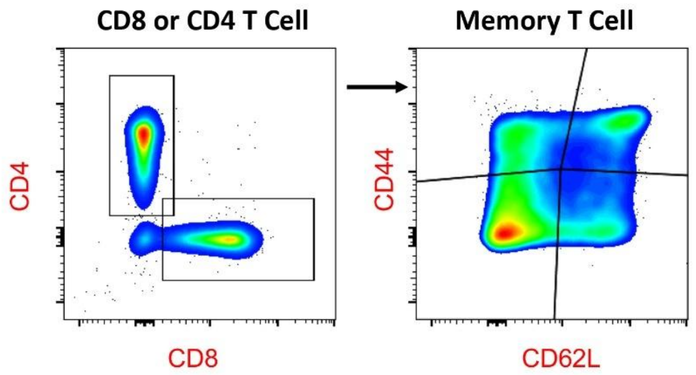

Supplementary Figure 44. Representative flow gating strategies for memory CD8 or CD4 T cells (Fig. 4i-j and Supplementary Fig. 29).

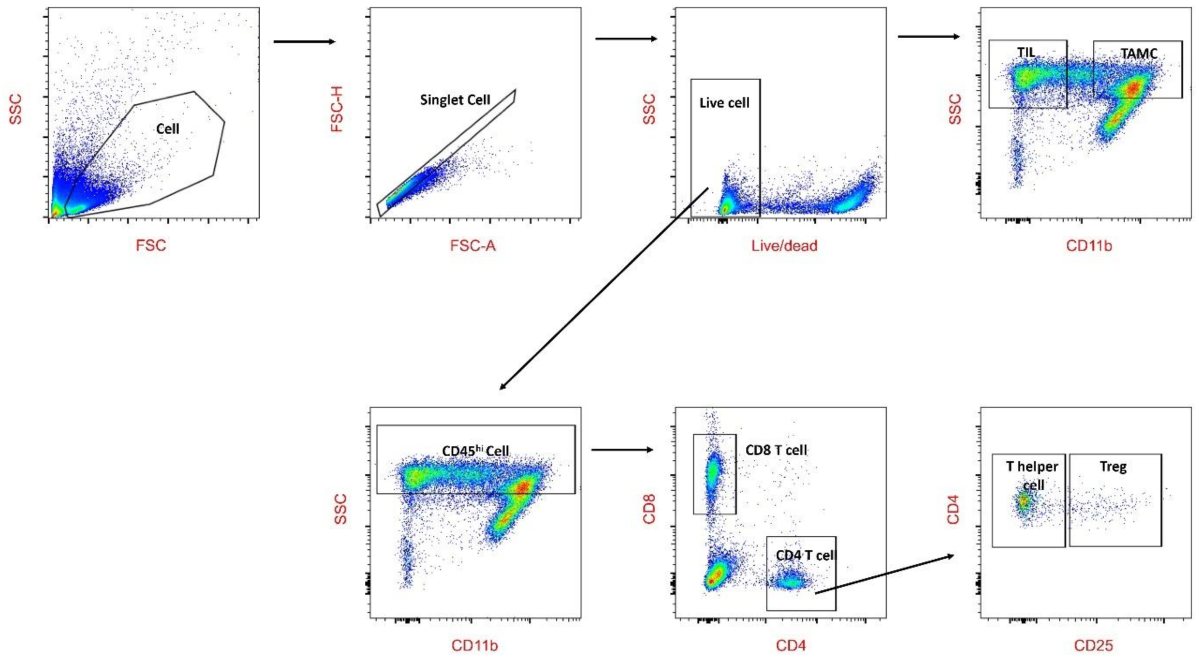

Supplementary Figure 45. Representative flow gating strategies for the CT-2A glioma studies (in Fig. 7).
