# Supplementary material for: Integrating antigen capturing nanoparticles and type 1 conventional dendritic cell therapy for in situ cancer immunization
Source: Nat Commun. 2025 May 16;16:4578. doi: 10.1038/s41467-025-59840-w (PMC12084569; doi:10.1038/s41467-025-59840-w)
Supplement: Supplementary file 2 — Reporting Summary [file 41467_2025_59840_MOESM2_ESM.pdf]

nature portfolio

Corresponding author(s): Zongmin Zhao
Last updated by author(s): Mar 28, 2025

# Reporting Summary

Nature Portfolio wishes to improve the reproducibility of the work that we publish. This form provides structure for consistency and transparency in reporting. For further information on Nature Portfolio policies, see our Editorial Policies and the Editorial Policy Checklist.

## Statistics

For all statistical analyses, confirm that the following items are present in the figure legend, table legend, main text, or Methods section.

| n/a | Confirmed | |
|---|---|---|
| ☐ | ☒ | The exact sample size (*n*) for each experimental group/condition, given as a discrete number and unit of measurement |
| ☐ | ☒ | A statement on whether measurements were taken from distinct samples or whether the same sample was measured repeatedly |
| ☐ | ☒ | The statistical test(s) used AND whether they are one- or two-sided<br>*Only common tests should be described solely by name; describe more complex techniques in the Methods section.* |
| ☐ | ☒ | A description of all covariates tested |
| ☐ | ☒ | A description of any assumptions or corrections, such as tests of normality and adjustment for multiple comparisons |
| ☐ | ☒ | A full description of the statistical parameters including central tendency (e.g. means) or other basic estimates (e.g. regression coefficient) AND variation (e.g. standard deviation) or associated estimates of uncertainty (e.g. confidence intervals) |
| ☐ | ☒ | For null hypothesis testing, the test statistic (e.g. *F*, *t*, *r*) with confidence intervals, effect sizes, degrees of freedom and *P* value noted<br>*Give P values as exact values whenever suitable.* |
| ☒ | ☐ | For Bayesian analysis, information on the choice of priors and Markov chain Monte Carlo settings |
| ☒ | ☐ | For hierarchical and complex designs, identification of the appropriate level for tests and full reporting of outcomes |
| ☒ | ☐ | Estimates of effect sizes (e.g. Cohen's *d*, Pearson's *r*), indicating how they were calculated |

*Our web collection on statistics for biologists contains articles on many of the points above.*

## Software and code

Policy information about availability of computer code

| | |
|---|---|
| Data collection | Zen Software for confocal images, Excel, Living image software (Aura from Spectral Instruments Imaging) for LagoX images, BD FACSDiva Software for flow cytometry. |
| Data analysis | All statistical analyzes were performed on Graphpad Prism (version 10). All flow cytometry data were analyzed on Flowjo (version 10). Confocal imaging data were analyzed on Zen Software (Version 3.9) and Image J. |

For manuscripts utilizing custom algorithms or software that are central to the research but not yet described in published literature, software must be made available to editors and reviewers. We strongly encourage code deposition in a community repository (e.g. GitHub). See the Nature Portfolio guidelines for submitting code & software for further information.

## Data

Policy information about availability of data

All manuscripts must include a data availability statement. This statement should provide the following information, where applicable:

- Accession codes, unique identifiers, or web links for publicly available datasets
- A description of any restrictions on data availability
- For clinical datasets or third party data, please ensure that the statement adheres to our policy

The mass spectrometry proteomics data have been deposited to the ProteomeXchange Consortium via the PRIDE partner repository with the dataset identifier PXD062399 at https://www.ebi.ac.uk/pride/archive/projects/PXD062399. The remaining data supporting the results in this study are available within the paper, Supplementary Information, and Source Data file. The raw numbers for charts and graphs are available in the Source Data file whenever possible. Any additional

requests for information can be directed to and will be fulfilled by the corresponding authors. Source data are provided with this paper.

## Research involving human participants, their data, or biological material

Policy information about studies with human participants or human data. See also policy information about sex, gender (identity/presentation), and sexual orientation and race, ethnicity and racism.

| | |
|---|---|
| Reporting on sex and gender | N/A |
| Reporting on race, ethnicity, or other socially relevant groupings | N/A |
| Population characteristics | N/A |
| Recruitment | N/A |
| Ethics oversight | N/A |

Note that full information on the approval of the study protocol must also be provided in the manuscript.

# Field-specific reporting

Please select the one below that is the best fit for your research. If you are not sure, read the appropriate sections before making your selection.

[x] Life sciences [ ] Behavioural & social sciences [ ] Ecological, evolutionary & environmental sciences

For a reference copy of the document with all sections, see nature.com/documents/nr-reporting-summary-flat.pdf

# Life sciences study design

All studies must disclose on these points even when the disclosure is negative.

| | |
|---|---|
| Sample size | Sample size were determined based on 1) power analysis (power of 0.80; effect size of 0.6; significance level of 0.05), 2) pilot studies, and 3) the basis of previous experimental experience. |
| Data exclusions | No data exclusion was performed. |
| Replication | The experiments were repeated at least two times, and the experimental findings were reproducible. |
| Randomization | All experimental mice and cells were randomly allocated into experimental groups. |
| Blinding | No formal blinding was used for the animal studies. The investigators were aware of the allocated groups for research needs. |

# Reporting for specific materials, systems and methods

We require information from authors about some types of materials, experimental systems and methods used in many studies. Here, indicate whether each material, system or method listed is relevant to your study. If you are not sure if a list item applies to your research, read the appropriate section before selecting a response.

### Materials & experimental systems

| n/a | Involved in the study | |
|---|---|---|
| [ ] | [x] | Antibodies |
| [ ] | [x] | Eukaryotic cell lines |
| [x] | [ ] | Palaeontology and archaeology |
| [ ] | [x] | Animals and other organisms |
| [x] | [ ] | Clinical data |
| [x] | [ ] | Dual use research of concern |
| [x] | [ ] | Plants |

### Methods

| n/a | Involved in the study | |
|---|---|---|
| [x] | [ ] | ChIP-seq |
| [ ] | [x] | Flow cytometry |
| [x] | [ ] | MRI-based neuroimaging |

## Antibodies

| | |
|---|---|
| Antibodies used | The following antibodies were used in this work.<br>• CD45-Alexa Fluor 700 (clone QA17A26, BioLegend, catalog #157616), 1:400-1:2000 dilution<br>• B220-APC (clone RA3-6B2, BioLegend, catalog #103211), 1:300 dilution<br>• CD11c-APC/Cy7 (clone N418, BioLegend, catalog #117324), 1:50 dilution |

- CD11c-PE, (clone N418, BioLegend, catalog #117308), 1:1000 dilution
- CD11c-BV650 (clone N418, BioLegend, catalog #117339), 1:80-1:200 dilution
- CD103-Alexa Fluor 700, (clone 2E7, BioLegend, catalog #121442), 1:200 dilution
- CD103-BV711(clone 2E7, BioLegend, catalog #121435), 1:100 dilution
- CD86-APC, (clone GL-1, BioLegend, catalog #105012), 1:400 dilution
- CD86-BV785 (clone GL-1, BioLegend, catalog #105043), 1:50-1:200 dilution
- MHCII-PB, (clone M5/114.15.2, BioLegend, catalog #107620), 1:1000 dilution
- MHCII-BV605 (clone M5/114.15.2, BioLegend, catalog #107639), 1:50 dilution
- CD80-FITC, (clone 16-10A1, BioLegend, catalog #104706), 1:250 dilution
- CD80-BV605 (clone 16-10A1, BioLegend, catalog #104729), 1:20-1:40 dilution
- CD11b-FITC (clone M1/70, BioLegend, catalog #101205), 1:100-1:1000 dilution
- F4/80-BV510 (clone BM8, BioLegend, catalog #123135), 1:10-1:200 dilution
- CD206-Percp-Cyanine5.5 (clone C068C2, BioLegend, catalog #141715), 1:10-1:200 dilution
- Gr-1-Pacific Blue (clone RB6-8C5, BioLegend, catalog #108430), 1:50 dilution
- CD49b-PE/Dazzle 594 (clone DX5, BioLegend, catalog #108924), 1:100-1:500 dilution
- CD3-PE/Fire 700 (clone 17A2, BioLegend, catalog #100272), 1:50 - 1:500 dilution
- CD4-Spark Blue 550 (clone GK1.5, BioLegend, catalog #100474), 1:100-1:400 dilution
- CD8a-Spark UV 387 (clone 53-6.7, BioLegend, catalog #100798), 1:100-1:1000 dilution
- IFN-γ-APC/Fire 750 (clone XMG1.2, BioLegend, catalog #505860), 1:100-1:200 dilution
- CD62L-PE/Cyanine5 (clone MEL-14, BioLegend, catalog #104410), 1:50-1:200 dilution
- CD44-BV570 (clone IM7, BioLegend, catalog #103037), 1:40 dilution
- CD25-PE/Cyanine7 (clone PC61, BioLegend, catalog #102016), 1:20-1:40 dilution
- FoxP3-Alexa Fluor 647 (clone MF-14, BioLegend, catalog #126408), 1:100-1:200 dilution
- PD-1-BV421 (clone 29F.1A12, BioLegend, catalog #135221), 1:100-1:200 dilution
- Adpgk tetramer-PE (Sequence: ASMTNMELM, Tetramer Core of the National Institutes of Health), 1:100 dilution
- OVA tetramer-PE (Sequence: SIINFEKL, Tetramer Core of the National Institutes of Health), 1:100 dilution
- TRP-2 tetramer-PE (Sequence: SVYDFFVWL, Tetramer Core of the National Institutes of Health), 1:100 dilution
- Rpl18 tetramer-BV421 (Sequence: KILTFDRL, Tetramer Core of the National Institutes of Health), 1:100 dilution
- Granzyme B-PE/Cy7 (clone QA16A02, BioLegend, catalog # 372213) 1:40 dilution
- Lag3-PE/Dazzle594 (clone C9B7W, BioLegend, catalog #125223) 1:80 dilution
- Tim3-PE/Fire640 (clone RMT3-23, BioLegend, catalog # 119749) 1:40-1:80 dilution
- PD1-PE/Fire810 (clone 29F.1A12, BioLegend, catalog #135253) 1:160 dilution
- Perforin-APC/Fire750 (clone S16009A, BioLegend, catalog #154317) 1:40-1:80 dilution
- Ki67-BV711 (clone 11F6, BioLegend, catalog #151227) 1: 300 dilution
- Clec9A-PE (clone 7H11, BioLegend, catalog #143504), 1:150 dilution
- H-2Kb-SIINFEKL-PE (clone 25-D1.16, BioLegend, catalog #141603), 1:160 dilution
- Zombie NIR (BioLegend, catalog #423106), 1:1000 dilution
- Zombie UV (BioLegend, catalog #423107), 1:1000 dilution

Validation

All antibodies were verified by the supplier, and each lot was quality-tested. All antibodies were titrated by the authors before being used for experiments.

## Eukaryotic cell lines

Policy information about cell lines and Sex and Gender in Research

Cell line source(s)

The MC38 cell line (Catalog # ENH204-FP) was purchased from Kerafast. The B16F10 cell line expressing luciferase (B16F10-Luc) (Catalog # CRL-6475-LUC2 ™), and B16F10 cell line (Catalog # CRL-6475) were obtained from ATCC. The B16F10-OVA cells were obtained as a gift from Dr. Darrel Irvine at Massachusetts Institute of Technology. The CT-2A cells were obtained as a gift from Dr. Tom Seyfried at Boston College. Primary dendritic cells were cultured from mouse bone marrow.

Authentication

All cell lines were authenticated by CLEAR PCR panels from Charles River Laboratory.

Mycoplasma contamination

All cell lines were tested for mycoplasma contamination. No mycoplasma contamination was found.

Commonly misidentified lines
(See ICLAC register)

No commonly misidentified cell lines were used.

## Animals and other research organisms

Policy information about studies involving animals; ARRIVE guidelines recommended for reporting animal research, and Sex and Gender in Research

Laboratory animals

Male/female C57BL/6 and female B6.129S(C)-Batf3tm1Kmm/J mice (6-8 weeks of age) and were purchased from Jackson Laboratories. Mice were housed in a facility with controlled conditions, including a 14:10-h light:dark cycle, an ambient temperature maintained at 22 ± 2 °C, and a relative humidity of 30–70%.

Wild animals

No wild animals were involved.

| | |
|---|---|
| Reporting on sex | Both male and female mice were used in the CT-2A tumor studies. Only female mice were used in the MC38 and B16F10 related tumor studies, due to challenges in housing a large number of male mice. |
| Field-collected samples | No field-collected samples were involved in this study. |
| Ethics oversight | All the animal experiments were performed in compliance with National Institutes of Health and institutional guidelines. All animal procedures were conducted according to approved protocols by the Institutional Animal Care and Use Committee (IACUC) at the University of Illinois Chicago (21-098, 24-085) and Northwestern University (IS00029388). |

Note that full information on the approval of the study protocol must also be provided in the manuscript.

## Plants

| | |
|---|---|
| Seed stocks | *Report on the source of all seed stocks or other plant material used. If applicable, state the seed stock centre and catalogue number. If plant specimens were collected from the field, describe the collection location, date and sampling procedures.* |
| Novel plant genotypes | *Describe the methods by which all novel plant genotypes were produced. This includes those generated by transgenic approaches, gene editing, chemical/radiation-based mutagenesis and hybridization. For transgenic lines, describe the transformation method, the number of independent lines analyzed and the generation upon which experiments were performed. For gene-edited lines, describe the editor used, the endogenous sequence targeted for editing, the targeting guide RNA sequence (if applicable) and how the editor was applied.* |
| Authentication | *Describe any authentication procedures for each seed stock used or novel genotype generated. Describe any experiments used to assess the effect of a mutation and, where applicable, how potential secondary effects (e.g. second site T-DNA insertions, mosiacism, off-target gene editing) were examined.* |

## Flow Cytometry

### Plots

Confirm that:

☒ The axis labels state the marker and fluorochrome used (e.g. CD4-FITC).

☒ The axis scales are clearly visible. Include numbers along axes only for bottom left plot of group (a 'group' is an analysis of identical markers).

☒ All plots are contour plots with outliers or pseudocolor plots.

☒ A numerical value for number of cells or percentage (with statistics) is provided.

### Methodology

| | |
|---|---|
| Sample preparation | The tissue was first made to single-cell suspension using tissue homogenization kit from Miltenyl Biotech or RWD Life Sciences, and single cells were resuspended in flow staining buffer for further processing. |
| Instrument | Beckman CytoFLEX, Cytek Aurora |
| Software | BD FACSDiva, SpectroFlo |
| Cell population abundance | In all experiments, at least 10,000 cells were counted and analyzed. |
| Gating strategy | Cells were first gated on FSC/SSC. Single cells were then gated using FSC-H and FSC-A. Live cells were then gated based on live/dead staining. Further gating was conducted on live single cells. |

☒ Tick this box to confirm that a figure exemplifying the gating strategy is provided in the Supplementary Information.
